# Deuterium Metabolic Imaging Differentiates Glioblastoma Metabolic Subtypes and Detects Early Response to Chemoradiotherapy

**DOI:** 10.1158/0008-5472.CAN-23-2552

**Published:** 2024-04-18

**Authors:** Jacob C.M. Low, Jianbo Cao, Friederike Hesse, Alan J. Wright, Anastasia Tsyben, Islam Alshamleh, Richard Mair, Kevin M. Brindle

**Affiliations:** 1 Cancer Research UK Cambridge Institute, University of Cambridge, Li Ka Shing Centre, Cambridge, United Kingdom.; 2 Division of Neurosurgery, Department of Clinical Neurosciences, University of Cambridge, Cambridge, United Kingdom.

## Abstract

Metabolic subtypes of glioblastoma (GBM) have different prognoses and responses to treatment. Deuterium metabolic imaging with ^2^H-labeled substrates is a potential approach to stratify patients into metabolic subtypes for targeted treatment. In this study, we used ^2^H magnetic resonance spectroscopy and magnetic resonance spectroscopic imaging (MRSI) measurements of [6,6′-^2^H_2_]glucose metabolism to identify metabolic subtypes and their responses to chemoradiotherapy in patient-derived GBM xenografts *in vivo*. The metabolism of patient-derived cells was first characterized *in vitro* by measuring the oxygen consumption rate, a marker of mitochondrial tricarboxylic acid cycle activity, as well as the extracellular acidification rate and ^2^H-labeled lactate production from [6,6′-^2^H_2_]glucose, which are markers of glycolytic activity. Two cell lines representative of a glycolytic subtype and two representative of a mitochondrial subtype were identified. ^2^H magnetic resonance spectroscopy and MRSI measurements showed similar concentrations of ^2^H-labeled glucose from [6,6′-^2^H_2_]glucose in all four tumor models when implanted orthotopically in mice. The glycolytic subtypes showed higher concentrations of ^2^H-labeled lactate than the mitochondrial subtypes and normal-appearing brain tissue, whereas the mitochondrial subtypes showed more glutamate/glutamine labeling, a surrogate for tricarboxylic acid cycle activity, than the glycolytic subtypes and normal-appearing brain tissue. The response of the tumors to chemoradiation could be detected within 24 hours of treatment completion, with the mitochondrial subtypes showing a decrease in both ^2^H-labeled glutamate/glutamine and lactate concentrations and glycolytic tumors showing a decrease in ^2^H-labeled lactate concentration. This technique has the potential to be used clinically for treatment selection and early detection of treatment response.

**Significance:**

Deuterium magnetic resonance spectroscopic imaging of glucose metabolism has the potential to differentiate between glycolytic and mitochondrial metabolic subtypes in glioblastoma and to evaluate early treatment responses, which could guide patient treatment.

## Graphical Abstract

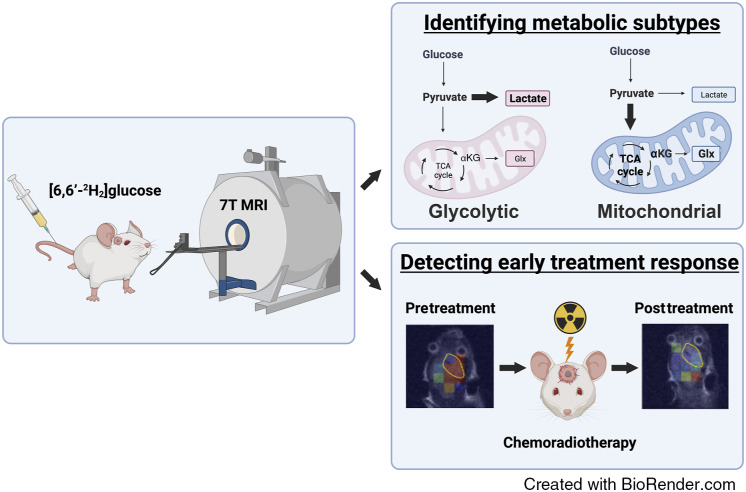

## Introduction

Metabolic reprogramming drives tumor cell proliferation and is an established hallmark of cancer (1, 2). Mutations in MYC and TP53 and mutations affecting signaling pathways, such as the PI3K/AKT/mTOR pathway (3), can result in the upregulation of tumor glucose metabolism and cellular energy production and the provision of intermediates for macromolecular biosynthesis. Tumor metabolic phenotypes that evolve as tumors progress and that present distinct metabolic vulnerabilities have been identified (4). A recent pathway-based computational analysis using single-cell and whole-tumor RNA sequencing (RNA-seq) data from isocitrate dehydrogenase wild-type glioblastoma (GBM) revealed four stable cellular states, two of which were distinct metabolic states with differing prognoses and therapeutic vulnerabilities (5): a mitochondrial subtype susceptible to therapies that inhibit oxidative phosphorylation, which has a better clinical outcome, and a glycolytic/plurimetabolic (GPM) subtype that has upregulated glycolytic, lipid, and amino acid metabolism, which is more resistant to radiotherapy and has a poorer prognosis. Although individual tumors can contain all four cellular states, most expressed a dominant state. The mitochondrial subtype is distributed across all three groups defined by previous transcriptomic classifications of GBM (6), whereas the glycolytic subtype is associated with the previously defined mesenchymal subtype. The development of tests to stratify patients into metabolic subtypes for targeted treatment has been identified as a clinical need (4, 7).

Metabolic subtypes can potentially be identified noninvasively using clinically applicable metabolic imaging techniques (4), which can also be used to detect early evidence of treatment response (8). The most widely available metabolic imaging technique in the clinic is PET with the glucose analog 2-deoxy-2-[^18^F]fluoro-D-glucose (^18^FDG), which can provide a measure of glucose uptake by facilitated glucose transporters but not necessarily glycolytic activity (9). However, a key limitation in brain tumors is the high uptake in normal surrounding brain tissue, which reduces tumor contrast. Nevertheless, this technique has been used in malignant gliomas, in which increased uptake prior to treatment and following first-line radiotherapy has been correlated with worse outcomes (10). Amino acid metabolism can be probed using ^11^C- and ^18^F-labeled amino acids. These provide better tumor contrast than ^18^FDG and are better for defining metabolically active GBM, providing prognostic information, and predicting progression-free survival and overall survival following chemoradiation (10). Advanced MRI techniques, including magnetic resonance spectroscopy (MRS) measurements of tumor metabolism, have also been shown to be superior to ^18^FDG-PET for determining prognosis and assessing treatment response (11). ^1^H MRS measurements of metabolism can distinguish true disease progression from pseudoprogression, with increases in choline signal with disease progression and decreases in choline signal and increases in lipid and lactate signals in posttreatment necrotic tumors (12). Treatment response can also be detected using hyperpolarized ^13^C-labeled pyruvate. The early response of orthotopically implanted patient-derived xenografts (PDX) to chemoradiation could be detected as a decrease in lactate labeling following an i.v. administration of hyperpolarized [1-^13^C]pyruvate (13). This technique also has the potential to identify metabolic subtypes of GBM. ^13^C magnetic resonance spectroscopic imaging (MRSI) measurements in a patient with GBM showed increased tumor lactate labeling when compared with the surrounding normal-appearing brain parenchyma (14), and a subsequent study showed heterogeneity between patients in the extent of lactate and bicarbonate labeling [bicarbonate is labeled via CO_2_, which is produced in the reaction catalyzed by pyruvate dehydrogenase in the tricarboxylic acid (TCA) cycle and is thus an indicator of mitochondrial activity; ref. 15]. This study mirrored a similar study in GBM PDXs, in which some PDXs showed high levels of lactate labeling, whereas others showed labeling that was no higher than that in the surrounding brain tissue (13). However, this technique is limited by the lifetime of the hyperpolarization, which in [1-^13^C]pyruvate is approximately 2 to 3 minutes *in vivo*, and the requirement for an expensive onsite polarizer, which is technically challenging to operate (16).

Deuterium metabolic imaging (DMI) with ^2^H-labeled substrates such as glucose (17, 18) and fumarate (19, 20) has emerged as a potential alternative to hyperpolarized ^13^C MRSI for imaging tissue metabolism (21, 22). DMI has been used to investigate glucose metabolism in patients with GBM following oral administration of [6,6′-^2^H_2_]glucose, in which higher lactate labeling and lower glutamate/glutamine (Glx) labeling were observed in the tumor than in the surrounding normal brain tissue (23). The low sensitivity of ^2^H detection is compensated for by its short T_1_ relaxation time, which allows for rapid signal averaging in the absence of signal saturation, and image resolutions comparable with those obtained with hyperpolarized ^13^C imaging have been achieved (24, 25), although the ^2^H images were acquired at a much higher field (9.4 T versus 3 T). In this study, we show that ^2^H MRS and MRSI measurements of [6,6′-^2^H_2_]glucose metabolism can differentiate between metabolic subtypes in GBM PDXs and can be used to detect response to chemoradiation within 24 hours of completion of this standard-of-care treatment.

## Materials and Methods

### Cell culture

Patient-derived isocitrate dehydrogenase wild-type GBM cell lines, A11 and S2, were obtained from Prof. Colin Watts and used at passages 15 and 8, respectively. AT5 cells were used at passage 12. The cells were derived at Addenbrooke’s Hospital using a protocol described previously (26). Tissue collection was approved by a Regional Ethics Committee (REC 18/EE/0283). Resected tumor samples were washed with Hank’s Balanced Salt Solution (HBSS; Gibco), minced using sterile razor blades, and enzymatically digested with Accutase (Sigma). Single cells were isolated by filtration through a 40-µm filter (Falcon). The cells were then centrifuged, and the pellet was incubated with 2 to 3 mL red blood cell lysis buffer (Sigma) for 5 minutes at room temperature. Patient-derived cells (A11, S2, and AT5) were seeded in extracellular matrix (Sigma)–coated flasks and grown as monolayer cultures in phenol red–free neurobasal A medium (Gibco) supplemented with 20 ng/mL human epidermal growth factor (hEGF; Sigma), 20 ng/mL human fibroblast growth factor (hFGF; Sigma), 2% B27 (Invitrogen), 1% N_2_ (Invitrogen), 2 mmol/L L-glutamine (Sigma), and 1% penicillin–streptomycin (Invitrogen). The human GBM cell line U87 (passage 79; ATCC, catalog no. HTB-14, RRID: CVCL_0022) was cultured in DMEM supplemented with 10% FBS (Gibco). When confluent, the cells were washed with HBSS, and patient-derived cells were detached using Accutase and U87 cells using 0.25% trypsin (Gibco). Cell counts and viability were determined using a Vi-CELL XR cell viability analyzer (Vi-CELL XR, Beckman Coulter; RRID: SCR_019664). Short tandem repeat profiling was performed using the PowerPlex_16HSM_cell line panel and analyzed using Applied Biosystems GeneMapper 5 software (RRID: SCR_021103; Thermo Fisher Scientific). This showed a 100% match to the in-house reference profiles when the lines were initially established (A11, S2, and AT5) or the Cellosaurus ST database (U87). All cells tested negative for *Mycoplasma* using the Phoenix qPCR Mycoplasma kit (Procomcure Biotech).

### Metabolic characterization of tumor cells *in vitro*

The baseline oxygen consumption rate (OCR) and extracellular acidification rate (ECAR) were measured using a Seahorse Bioscience XF 96 analyzer (RRID: SCR_019545, Agilent). A total of 45,000 cells per well were seeded in a 96-well plate 24 hours prior to the assay. On the day of the assay, supplemented neurobasal medium for patient-derived cells or DMEM for U87 cells were replaced with Seahorse XF DMEM media and incubated for 1 hour in a CO_2_-free incubator. OCR and ECAR measurements were obtained for each tumor cell line at 5-minute intervals with seven to nine technical replicates. To normalize for cell count, at the end of each assay, the 96-well plate was incubated in 25 mmol/L Hoechst stain solution (Thermo Fisher), and fluorescence was read using a plate reader (CLARIOstar Plus, BMG Labtech). Three to six biological replicates were obtained for each tumor cell line. The data were processed using Seahorse Analytics (Agilent).

### ^2^H nuclear magnetic resonance and ^1^H-^13^C HSQC nuclear magnetic resonance spectroscopy of cell and media extracts

For the analysis of media extracts, patient-derived cells (1 × 10^6^) were seeded in extracellular matrix–coated T25 flasks and U87 cells (1 × 10^6^) in noncoated T25 flasks. For the analysis of cell extracts, the cells were grown in T75 flasks. The cells were grown for 5 days, and when the medium was removed, the cells were washed with HBSS and incubated either for 4 hours with 10 mmol/L of [6,6′-^2^H_2_]glucose (Sigma) in 5 mL of glucose-free neurobasal medium (for A11, S2, and AT5 cells) or glucose-free DMEM (for U87 cells) or for 6 hours with 10 mmol/L of [U-^13^C]glucose (Cambridge Isotope) in 10 mL of glucose-free neurobasal medium (for A11, S2, and AT5 cells) or glucose-free DMEM (for U87 cells). The longer incubation time with [U-^13^C]glucose was used to increase the concentrations of labeled lactate and Glx and, therefore, the signal-to-noise ratio in the heteronuclear single quantum coherence (HSQC) spectra. Medium samples were collected from the cells incubated with [6,6′-^2^H_2_]glucose. The cells were then detached, counted, and assessed for viability using the Vi-CELL XR cell viability analyzer. For cells incubated with [U-^13^C]glucose, flasks were immediately placed on ice following the 6-hour incubation period before the cells were detached and then extracted in chloroform/methanol (1:1, vol/vol; ref. 27). Medium samples and the aqueous fraction of the cell extracts were concentrated (Savant SpeedVac, Thermo Scientific) and dissolved in either 600 μL of PBS or in 600 μL of deuterium oxide, respectively. Five mmol/L ^2^H sodium formate (Sigma) was added to the medium samples, or 5 mmol/L sodium 3-trimethylsilyl-2,2,3,3-d_4_-propionate (TMSP; Cambridge Isotope) was added to the cell extracts. Spectra were acquired at 14.1 T (Bruker Spectrospin Ltd.) and 300 K. ^2^H spectra were acquired using the ^2^H coil of a 5-mm ^1^H/broadband inverse detection probe with a 90 degrees pulse and a 3-second repetition time. Spectra were measured as the sum of 1,024 transients collected over 62 minutes with a spectral width of 2,000 Hz. Metabolite concentrations were calculated by correcting for the T_1_ of formate and the number of deuterons. ^1^H-^13^C HSQC spectra were acquired into 1 K time domain points, with a 1.5-second repetition time, 16 dummy scans, 128 averages with a spectral width of 13.35 ppm for ^1^H and 110 ppm for ^13^C, and a total acquisition time of 12.5 hours. ^1^H and ^13^C chemical shifts were referenced to TMSP at 0.00 and 110.00 ppm, respectively. Chemical shifts were confirmed by spiking samples with 5 mmol/L of [U-^13^C]glucose (Cambridge Isotope), [5-^13^C]glutamate (Sigma-Aldrich), and [U-^13^C]lactate (Cambridge Isotope). Peak intensities were normalized to that of TMSP. Data analysis was performed using TopSpin software (Bruker Spectrospin Ltd.; RRID: SCR_014227). Metabolite concentrations were normalized to the corresponding cell counts.

### Orthotopic tumor implantation

Procedures were performed in compliance with personal and project licenses issued under the United Kingdom Animals Scientific Procedures Act (1986) and approved by the Cancer Research UK, Cambridge Institute Animal Welfare, and Ethical Review Body. Twelve-week-old female BALB/c nude mice (Charles River Laboratories, UK; RRID: SCR_003792) weighing a minimum of 20 g were anesthetized by inhalation of 1% isoflurane (Abbott Laboratories) in air/oxygen (75%/25%) at a flow rate of 1 L/minute. Subcutaneous analgesia (0.3 mg/mL buprenorphine hydrochloride and 0.135% w/v chlorocresol diluted 1:10 in 0.9% sodium chloride and 1 mL/kg of Rimadyl (Pfizer) diluted 1:10 in 0.9% sodium chloride) was provided prior to implantation. The perioperative respiration rate was monitored, and the body temperature was maintained using a heated pad. The animals were positioned in a stereotactic surgical frame (Kopf), and the head was secured using bite and ear bars. The head was cleaned with 4% aqueous chlorhexidine gluconate (Ecolab), and a midline incision was made. The pericranium was stripped, and a 1-mm burr hole was drilled 3 mm lateral (right) and 2 mm anterior to the bregma. A Hamilton needle (Sigma) was filled with 5 µL of cell suspension (0.3 × 10^6^ cells/µL) and passed through the right frontal lobe of the brain at a depth of 3.5 mm. The needle was withdrawn (0.5 mm), and the cell suspension was injected at 2 µL/minute. The burr hole was replaced with bone wax (Ethicon), and the wound was sutured with 6/0 Vicryl (Ethicon) and reinforced with tissue glue (GLUture). The animals were recovered in a warm box and received postoperative subcutaneous Rimadyl every 24 hours for 48 hours.

### Image-guided targeted cranial irradiation

Tumors were treated when they were >70 mm^3^. One hour prior to irradiation, the animals received 100 mg/kg temozolomide (Cambridge Bioscience) by oral gavage. Animals were anesthetized with isoflurane and positioned within a small animal radiation research platform device (XStrahl, Walsall) before receiving 5 Gy of image-guided targeted radiotherapy to the right frontal lobe, as described previously (20).

### ^1^H MRI

Following orthotopic implantation, tumor growth was monitored at 9.4 T (Bruker; RRID: SCR_018054) using a 40-mm i.d. Millipede ^1^H volume coil (Agilent). The mice were anesthetized by inhalation of 1% isoflurane in air/oxygen (75%/25%) at a flow rate of 1 L/minute and positioned using a bite bar. While anesthetized and undergoing imaging, vital signs and temperature were monitored (Small Animal Instruments; RRID: SCR_002090). T_2_-weighted axial and coronal images were acquired using a fast spin echo pulse sequence with a repetition time of 2 seconds, echo time of 40 milliseconds, data matrix of 256 × 256 points, field of view (FOV) 32 × 32 mm^2^, slice thickness of 1 mm; 9 slices, and 4 averages. Tumors were delineated using ImageJ (NIH; RRID: SCR_003070), and volumes were calculated by summing the areas of successive slices through the tumor.

### ^2^H MRS *in vivo*

Coil-localized ^2^H spectra were acquired when tumors were >70 mm^3^ (73.32 mm^3^ ± 2.51 mm^3^, *n* = 22) using a 7 T scanner (Agilent) with a custom-built 14-mm single-loop ^2^H transmit–receive coil and ^1^H volume transmit–receive coil (RAPID Biomedical). Spectra were also acquired from tumor-free mice (*n* = 4). Animals were anesthetized, positioned, and monitored as described above. Tail vein cannulation was performed prior to imaging. Sequential coil-localized ^2^H spectra were acquired with a spectral width of 2,003 Hz into 256 data points over a period of 65 minutes using a 2 milliseconds BIR4 pulse with a nominal flip angle of 50 degrees, TR of 140 milliseconds, and 2,250 averages. After acquisition of a baseline spectrum, [6,6′-^2^H_2_]glucose (Sigma) dissolved in saline (0.2 g/mL) was injected over 10 minutes using an infusion pump (Harvard Apparatus) to obtain a final concentration of 2 g/kg body weight. Tumor-bearing animals then underwent a 4-day regimen of oral temozolomide, dissolved in water (100 mg/kg/day), and 5 Gy/day of image-guided targeted radiation delivered before repeat ^2^H MRS measurements within 24 hours of the last day of treatment.

Spectra were zero- and first-order phase-corrected, and resonances were fitted using a routine based on the AMARES toolbox in MATLAB (MATLAB; RRID: SCR_001622) in which the line shapes were restricted to be Gaussian. The following chemical shifts were used: water, 4.7 ppm; glucose, 3.72 ppm; Glx, 2.3 ppm; and lactate, 1.3 ppm. The concentrations of deuterated metabolites in the brain were calculated by normalizing the fitted resonance integrals to the baseline water signal [measured to be 12.48 mmol/L ^2^H-labeled water (HDO) in Cambridge, assuming a brain water content of 80%; ref. 28] and corrected for the number of deuterons in each metabolite and for signal saturation, assuming the following T_1_ relaxation times: water, 320 milliseconds; glucose, 64 milliseconds; Glx, 146 milliseconds; and lactate, 297 milliseconds (23).

### ^2^H three-dimensional chemical shift imaging *in vivo*

^2^H three-dimensional (3D) chemical shift images were acquired at 7 T when tumors were >70 mm^3^. Images were also acquired from tumor-free mice (*n* = 3). Tumors were first localized in coronal ^1^H images acquired using a T_2_-weighted fast spin echo pulse sequence: TR, 2 seconds; echo time, of 48 milliseconds; FOV, 27 × 27 mm^2^, 128 × 128 matrix; and slice thickness, 3 mm; 9 slices. After acquisition of a baseline image, [6,6′-^2^H_2_]glucose (Sigma) was injected to obtain a final concentration of 2 g/kg. Serial ^2^H chemical shift imaging (CSI) spectra were acquired using a 2 milliseconds BIR4 pulse with a nominal flip angle of 50 degrees, with phase-encoding gradients encoding a 9 × 9 × 3 k-space matrix with an FOV of 27 × 27 × 27 mm. Data were acquired into 256 spectral points, with a spectral width of 2,003 Hz and a TR of 140 milliseconds. Metabolite concentrations were determined from a single voxel that the ^1^H images showed was entirely within the tumor and that had minimal contamination from surrounding brain tissue. Tensor denoising was performed, as described previously (18), reducing the dataset from 9 × 9 × 3 × 256 × 7 to 6 × 6 × 3 × 24 × 5. Spectra were phase-corrected, and the resonances were fitted, as described above.

### Histology and IHC

The brains were transferred to 10% formalin for 24 hours and then to 70% ethanol before embedding in paraffin and sectioning into 10-μm-thick sections, which were then stained with hematoxylin and eosin (ST020 multistainer; Leica Microsystems; RRID: SCR_008960). Ki67 staining and caspase 3 (CC3) staining were performed using a Leica Polymer Refine kit (Leica Microsystems; catalog no. DS9800; RRID: AB_2891238) on an automated bond platform (Leica Biosystems). The Ki67 antibody (M7240, Agilent; RRID: AB_2142367) was used at a 1:200 dilution, and the CC3 antibody (9664, Cell Signaling Technology; RRID: AB_2070042) was used at a 1:400 dilution. The slides were scanned at 20× magnification with a 0.5 μm per pixel resolution on an Aperio AT2 scanner (Leica Biosystems; RRID: SCR_021256). Images were analyzed using a CytoNuclear v1.6 algorithm on HALO (Indica Labs; RRID: SCR_018350) to quantify the percentage of positive cells.

### Statistical analysis

Statistical and graphical analyses were performed using Prism v9 (GraphPad; RRID: SCR_002798). Data are shown as the mean ± SD unless stated otherwise. ANOVA was performed for multiple comparisons of groups to determine significance. A paired Student *t* test was used for single-parameter comparisons.

### Data availability

The data presented in each of the figures are available in the form of Excel spreadsheets at https://doi.org/10.17863/CAM.107222. All raw data generated in this study are available upon request from the corresponding author.

## Results

### Metabolic characterization of GBM cells *in vitro*

**Figure 1.**
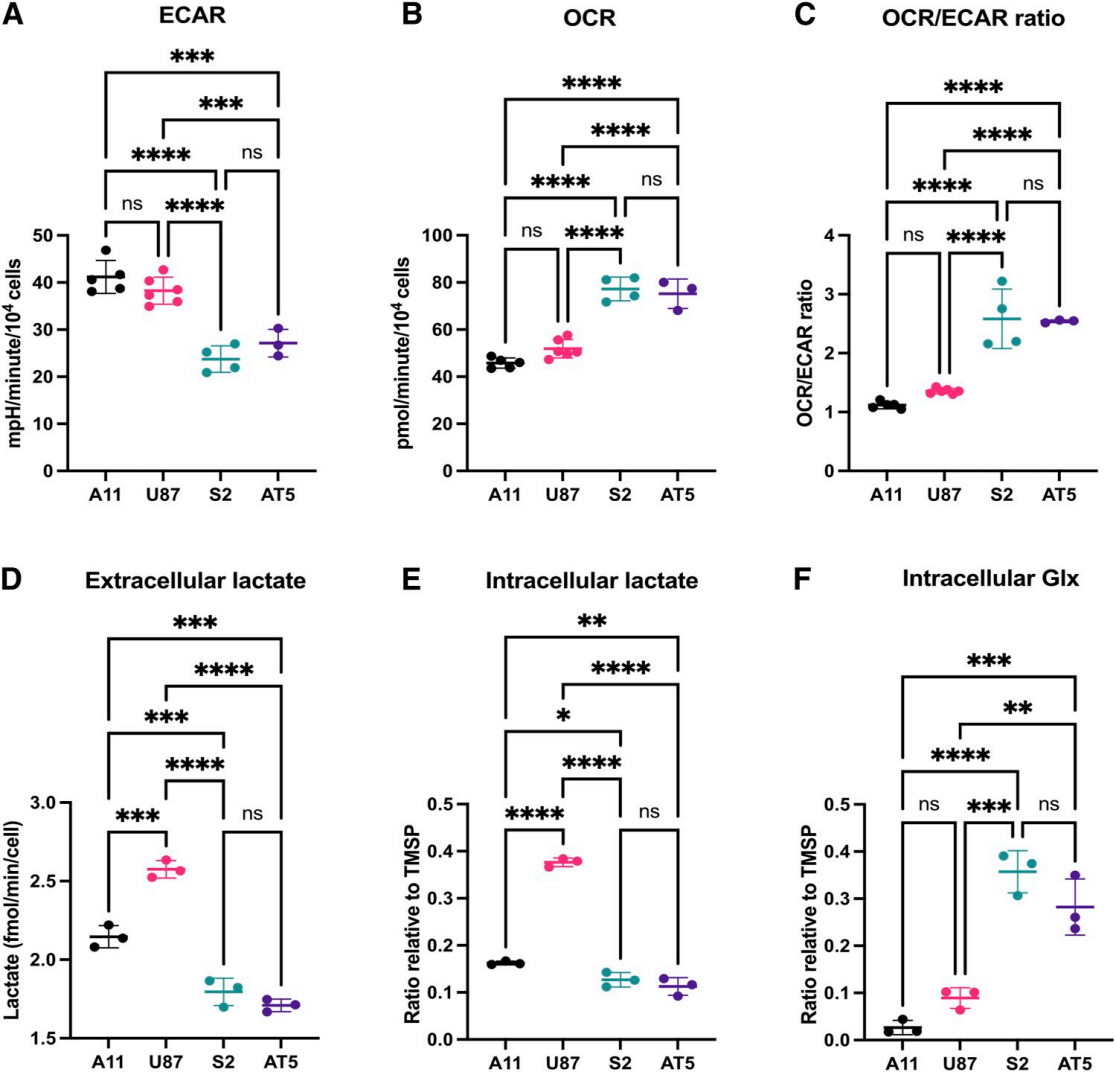
Metabolic characterization of GBM tumor cells *in vitro*. **A,** ECAR measurements. There were no significant differences between A11 and U87 cells (*P* = 0.427) or between S2 and AT5 cells (*P* = 0.494). **B,** OCR measurements. There were no significant differences between S2 and AT5 cells (*P* = 0.920) or between A11 and U87 cells (*P* = 0.127). **C,** OCR/ECAR ratio. There were no significant differences between S2 and AT5 cells (*P* = 0.996) or between A11 and U87 cells (*P* = 0.378). **D,**^2^H MRS measurements of ^2^H-labeled lactate produced by A11, S2, and AT5 cells incubated for 4 hours in glucose-free neurobasal media and U87 cells incubated in glucose-free DMEM, both supplemented with 10 mmol/L [6,6′-^2^H_2_]glucose. **E** and **F,** HSQC ^1^H-^13^C MRS measurements of ^13^C-labeled lactate (**E**) and Glx (**F**) measured in cell extracts obtained by chloroform–methanol extraction following incubation for 6 hours in glucose-free media supplemented with 10 mmol/L [U-^13^C]glucose. Proton signal intensities in the spectra are reported relative to the TMSP standard. ns, not significant; *, *P* < 0.05; **, *P* < 0.01; ***, *P* < 0.001; ****, *P* < 0.0001.

A11 and U87 cells displayed a glycolytic phenotype with ECARs that were significantly greater than those displayed by S2 and AT5 cells (Fig. 1A), whereas S2 and AT5 cells displayed a more oxidative phenotype with basal OCRs that were significantly greater than those displayed by A11 and U87 cells (Fig. 1B). The average OCR/ECAR ratios were significantly higher in S2 (2.6 ± 0.5) and AT5 (2.5 ± 0.02) cells than in A11 (1.1 ± 0.06) and U87 (1.4 ± 0.05) cells (Fig. 1C). Consistent with the ECAR measurements, ^2^H MRS measurements of medium samples from cells incubated with 10 mmol/L [6,6′-^2^H_2_]glucose for 4 hours showed that A11 and U87 cells produced significantly more ^2^H-labeled lactate than AT5 and S2 cells (Fig. 1D). HSQC ^1^H-^13^C nuclear magnetic resonance (NMR) measurements of extracts of cells incubated with [U-^13^C]glucose showed higher concentrations of intracellular ^13^C-labeled lactate and lower concentrations of ^13^C-labeled glutamate in A11 and U87 cells compared with those in AT5 and S2 cells (Fig. 1E and F). The relative concentrations of ^13^C-labeled lactate in the different cell lines paralleled their relative rates of ^2^H-labeled lactate production. Representative HSQC ^1^H-^13^C NMR spectra are shown in Supplementary Fig. S1A–S1D. Analysis of RNA-seq data obtained previously from A11 and S2 tumors implanted orthotopically in rats (29), using the pathway-level classification of Garofano and colleagues (5), showed that A11 belongs to the GPM subgroup, whereas S2 belongs to the mitochondrial subgroup (Supplementary Fig. S2). A previous analysis of these RNA-seq data showed that A11 belongs to a mesenchymal subgroup (29), which is positively associated with the GPM subgroup (5).

### Deuterated glucose metabolism differentiates metabolic subtypes of GBM cells *in vivo*

**Figure 2.**
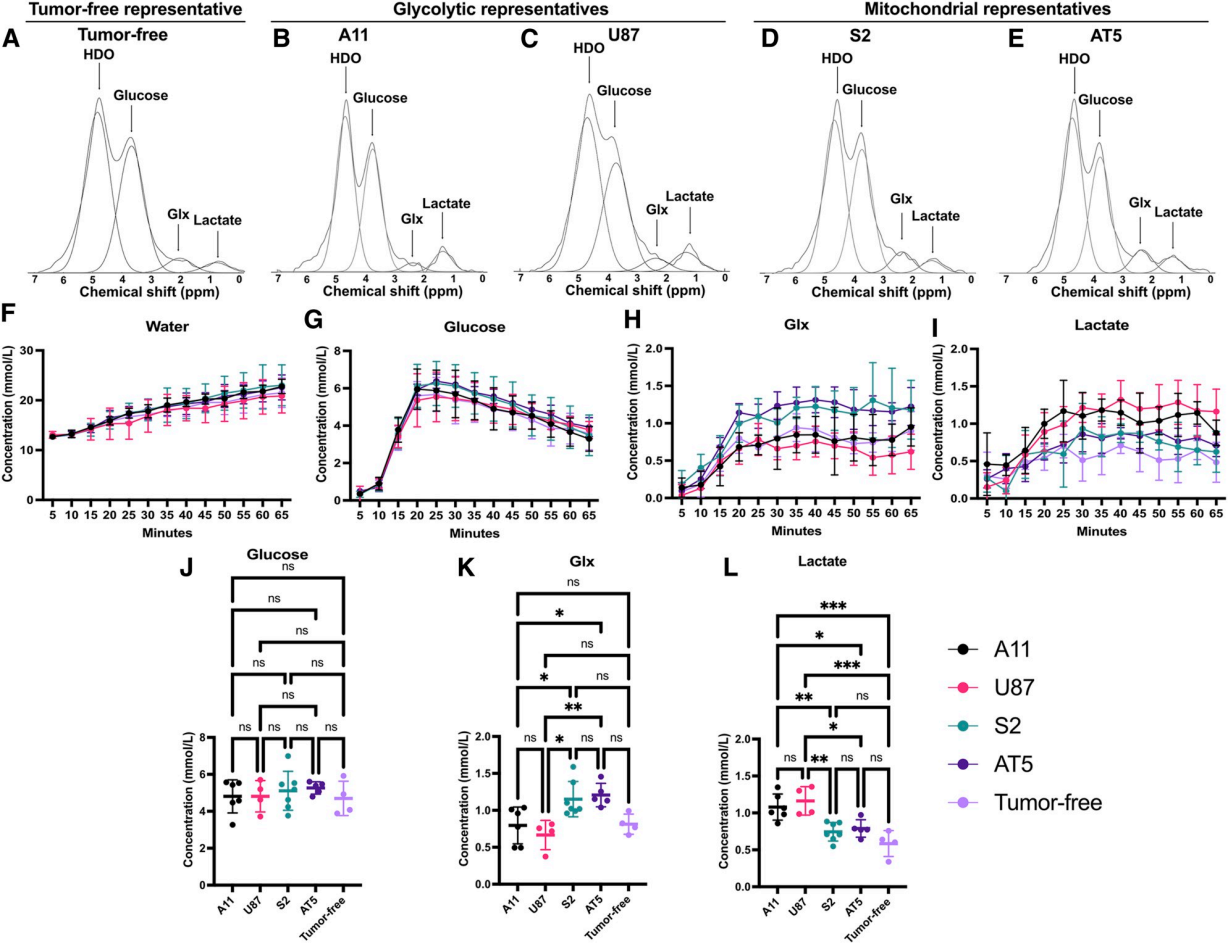
Coil-localized ^2^H MRS differentiates metabolic subtypes of GBM *in vivo*. **A-E,** Representative summed 5-minute ^2^H spectra from tumor-free mice (**A**) and tumor-bearing mice with A11 (**B**), U87 (**C**), S2 (**D**), and AT5 tumors (**E**) acquired over a period of 60 minutes following an i.v. injection of 2 g/kg [6,6′-^2^H_2_]glucose. The summed spectra overlaid with the corresponding peak fits are shown. **F-I,** Concentrations of HDO (**F**), glucose (**G**), Glx (**H**), and lactate (**I**) determined from the fitted peaks in individual 5-minute spectra. **J-L,** The average concentrations of ^2^H-labeled glucose (**J**), Glx (**K**), and lactate (**L**) measured between 20 and 65 minutes following injection of [6,6′-^2^H_2_] glucose in the four tumor models and in tumor-free animals. A one-way ANOVA showed that there were no significant differences in the labeled glucose concentration in the four tumor models and in tumor-free mice, but there were significant differences in the concentrations of labeled lactate and Glx between the glycolytic and mitochondrial subtype tumors and between the concentrations of labeled lactate in the glycolytic subtype tumors and the tumor-free animals. ns, not significant; *, *P* < 0.05; **, *P* < 0.01; ***, *P* < 0.001.

Mice were implanted orthotopically with A11 (*n* = 6), U87 (*n* = 4), S2 (*n* = 7), or AT5 (*n* = 5) cells. When tumors reached a volume greater than 70 mm^3^, coil-localized ^2^H MRS spectra were acquired in 5-minute blocks following an i.v. injection of 2 g/kg of [6,6′-^2^H_2_]glucose. Spectra were also acquired from non–tumor-bearing animals (*n* = 4). Representative 5-minute spectra are shown in Supplementary Fig. S3, and the summed spectra acquired over a period of 65 minutes are shown in Fig. 2A–E. HDO, glucose, Glx, and lactate concentrations measured at 5-minute intervals are shown in Fig. 2F−I. The Glx concentration was determined from the unresolved glutamate and glutamine resonances. The concentrations of labeled glucose in the tumors and in the normal brain were similar, peaking at 20 minutes following injection and declining thereafter. The concentrations of ^2^H-labeled glucose, lactate, and Glx were calculated from 10 summed spectra acquired between 20 and 65 minutes following glucose injection. There were no significant differences in the concentration of ^2^H-labeled glucose between the four tumor models and between the tumor models and normal brain (*P* = 0.83; Fig. 2J). The concentration of ^2^H-labeled Glx was not different between the glycolytic subtype (A11 vs. U87, *P* = 0.87) and mitochondrial subtype tumors (S2 vs. AT5, *P* = 0.99), but the mitochondrial subtype tumors had significantly higher concentrations of ^2^H-labeled Glx than the glycolytic subtype tumors (Fig. 2K). The concentrations of ^2^H-labeled Glx in tumor-free mice were not significantly different from the glycolytic subtype tumors, although they seemed to be lower when compared with the mitochondrial subtype tumors, but this was not significant (Fig. 2K). However, when the comparison was restricted to the mitochondrial subtype tumors, both S2 (*P* = 0.03) and AT5 tumors (*P* = 0.02) showed significantly higher concentrations of labeled Glx compared with the tumor-free mice (Supplementary Fig. S4A–S4C). The concentration of ^2^H-labeled lactate was not different between the glycolytic subtype tumors (A11 vs. U87, *P* = 0.92) or between the mitochondrial subtype tumors (S2 vs. AT5, *P* = 0.99); however, the glycolytic subtype tumors had significantly higher ^2^H-labeled lactate concentrations than the mitochondrial subtype tumors and normal brain in the tumor-free animals (Fig. 2L).

The initial rates of ^2^H-labeled Glx and lactate production were calculated for the first 20 minutes following ^2^H-labeled glucose injection. The initial rate of lactate production was 0.12 ± 0.03 mmol/L/minute in A11 tumors, which was similar to that in U87 tumors (0.11 ± 0.03 mmol/L/minute). These rates were significantly higher than those observed in tumor-free mice (0.08 ± 0.01 mmol/L/minute, *P* = 0.01). S2 and AT5 tumors also showed similar rates of ^2^H-labeled lactate production of 0.08 ± 0.01 and 0.08 ± 0.02 mmol/L/minute, respectively, but these were significantly lower than those in the glycolytic subtypes A11 and U87 (*P* = 0.0027) and not significantly different from the rate observed in tumor-free animals (*P* = 0.82). The rates of ^2^H-labeled Glx production were similar in S2 (0.12 ± 0.02 mmol/L/minute) and AT5 tumors (0.13 ± 0.02 mmol/L/minute) and in A11 (0.08 ± 0.02 mmol/L/minute) and U87 (0.08 ± 0.03 mmol/L/minute) tumors. However, the mitochondrial subtypes had significantly higher rates of ^2^H-labeled Glx production than the glycolytic subtypes (*P* = 0.0004). Tumor-free mice showed a significantly lower rate of ^2^H-labeled Glx production (0.09 ± 0.02 mmol/L/minute) compared with mitochondrial subtype tumors (*P* = 0.03) but not compared with the glycolytic subtypes (*P* = 0.67; Supplementary Fig. S5A and S5B).

In summary, tumors derived from cells that showed a glycolytic metabolic phenotype *in vitro* (A11 and U87) had higher concentrations of ^2^H-labeled lactate and lower levels of ^2^H-labeled Glx than tumors derived from cells that showed a mitochondrial metabolic phenotype (S2 and AT5).

### ^2^H 3D CSI differentiates tumor from the normal brain and distinguishes glycolytic and mitochondrial subtypes

**Figure 3.**
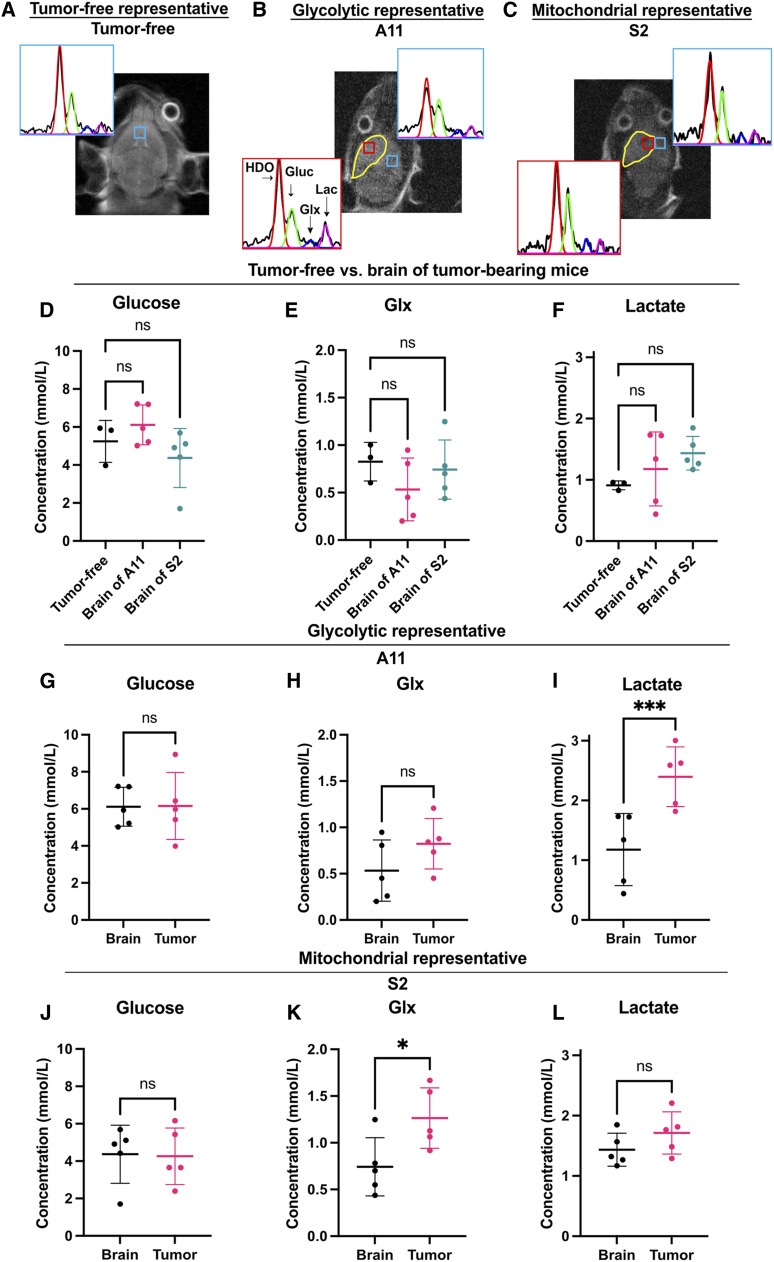
3D ^2^H CSI of [6,6-^2^H_2_]glucose metabolism in A11 and S2 tumors, in normal-appearing brain tissue, and in brain tissue in tumor-free mice. **A-C, **Representative spectrum from a voxel within the brain of a tumor-free mouse (**A**), and representative spectra from voxels within the tumor and normal-appearing brain of A11 (**B**) and S2 (**C**) tumor–bearing animals injected with 2 g/kg [6,6-^2^H_2_]glucose. Fits to the spectra are shown in color: red, water; green, glucose; blue, Glx; and purple, lactate. The tumor is outlined in yellow on the T_2_-weighted ^1^H image. The locations of the CSI voxels are indicated by the boxes. The spectra are the sum of six 10-minute spectra acquired from 3 × 3 × 9 mm voxels.** D-F,** Comparison of ^2^H-labeled glucose (**D**), Glx (**E**), and lactate (**F**) concentrations in the brains of tumor-free mice and the normal-appearing brain of mice bearing A11 and S2 tumors. **G-I,** Comparison of ^2^H-labeled glucose (**G**), Glx (**H**), and lactate (**I**) concentrations in A11 tumors and adjacent normal-appearing brain tissue. **J-L,** Comparison of ^2^H-labeled glucose (**J**), Glx (**K**), and lactate (**L**) concentrations in S2 tumors and adjacent normal-appearing brain tissue. ns, not significant; *, *P* < 0.05; ***, *P* < 0.001.

In a separate cohort of animals, we acquired 3D ^2^H chemical shift images following an i.v. injection of 2 g/kg [6,6′-^2^H_2_]glucose from tumor-free mice (*n* = 3) and from animals with A11 (*n* = 5) and S2 (*n* = 5) tumors. Representative spectra from a voxel in the brain of a tumor-free mouse and from voxels in the normal-appearing brain and tumor in A11 and S2 tumor–bearing mice are shown in Fig. 3A–C. Representative spectra from the 12 voxels that covered the entire brains of a tumor-free animal and of animals with A11 and S2 tumors are shown in Supplementary Fig. S6A–S6C. The concentrations of ^2^H-labeled glucose, Glx, and lactate in the normal-appearing brain of tumor-bearing animals were not significantly different from those in the brains of tumor-free mice (Fig. 3D–F). In A11 tumors, there were no significant differences between glucose (Fig. 3G) or Glx (Fig. 3H) concentrations and those in the normal-appearing brain; however, the ^2^H-labeled lactate concentration was significantly higher (Fig. 3I; *P* = 0.0003). In S2 tumors, the glucose concentration was also similar to that in the normal brain (Fig. 3J), whereas the ^2^H-labeled Glx concentration was significantly higher (Fig. 3K; *P* = 0.013), but the lactate concentration was similar to that in the normal brain (Fig. 3L). The concentration of labeled lactate was significantly higher in A11 tumors when compared with that in S2 tumors, whereas the concentration of labeled Glx was significantly lower (Supplementary Fig. S7). These data confirmed those obtained using coil-localized ^2^H MRS spectra and showed that a tumor belonging to the glycolytic metabolic subtype (A11) showed higher lactate labeling and lower Glx labeling *in vivo* than a tumor belonging to the mitochondrial subtype (S2).

### Detection of response to chemoradiotherapy

**Figure 4.**
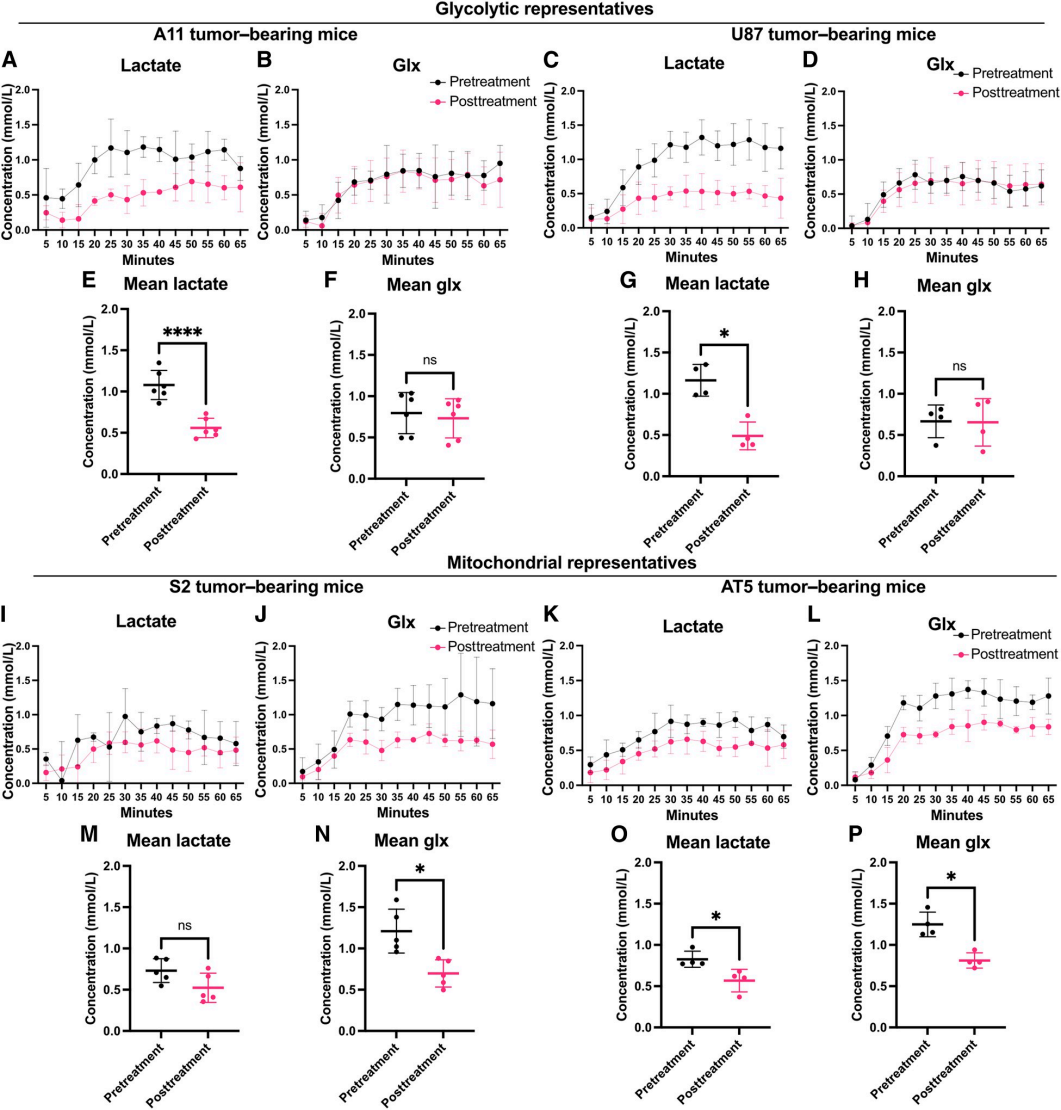
Spectroscopic measurements of the effects of chemoradiation on glucose metabolism in tumor models representative of the glycolytic and mitochondrial subtypes. Serial 5-minute tumor ^2^H spectra were acquired following an i.v. injection of [6,6′-^2^H_2_]glucose in two glycolytic subtypes of GBM (A11 and U87; **A–H**) and two mitochondrial subtypes (S2 and AT5; **I–P**) before and 24 hours after targeted chemoradiotherapy. Measurements of ^2^H-labeled lactate and Glx in serial 5-minute spectra acquired from tumor-bearing animals pre- and posttreatment of A11 (**A** and **B**), U87 (**C** and **D**), S2 (**I** and **J**), and AT5 (**K** and **L**) tumors. The concentrations of ^2^H-labeled lactate and Glx measured between 20 and 65 minutes following [6,6′-^2^H_2_]glucose injection were compared pre- and posttreatment in A11 (**E** and **F**), U87 (**G** and **H**), S2 (**M** and **N**), and AT5 (**O** and **P**) tumor–bearing animals. ns, not significant; *, *P* < 0.05; ****, *P* < 0.0001.

Pretreatment coil-localized ^2^H spectra were acquired from A11 and U87 tumors following the infusion of 2 g/kg [6,6-^2^H_2_]glucose. The animals then underwent a daily treatment regimen of 100 mg/kg of oral temozolomide, followed by 5 Gy of image-guided radiotherapy for 4 days, after which, posttreatment tumor spectra were acquired (Fig. 4A–D). In the glycolytic subtype A11 tumors, there was a 48% reduction in the labeled lactate concentration following treatment, from 1.08 ± 0.18 to 0.56 ± 0.12 mmol/L (*P* < 0.0001; *n* = 6; Fig. 4E), but no significant change in the Glx concentration was observed (Fig. 4F; *P* = 0.46). Similar changes were observed in U87 tumors (*n* = 4), in which there was a 58% reduction in the labeled lactate concentration, from 1.16 ± 0.19 mmol/L in the pretreatment animals to 0.49 ± 0.17 mmol/L in the treated animals (Fig. 4G), but no significant change in the Glx concentration was observed (Fig. 4H; *P* = 0.88).

Pre- and posttreatment coil-localized ^2^H spectra were also acquired from S2 and AT5 tumors, which are representative of the mitochondrial subtype (Fig. 4I–L). In S2 tumors, there was a nonsignificant decrease of 29% in the labeled lactate concentration, from 0.73 ± 0.14 to 0.52 ± 0.18 (*P* = 0.07, *n* = 5, Fig. 4M), and unlike in the glycolytic subtype tumors, a significant decrease of 42% in the labeled Glx concentration, from 1.21 ± 0.27 to 0.70 ± 0.17 mmol/L (*P* = 0.017; *n* = 5; Fig. 4N). In AT5 tumors, there was a decrease of 31% in the labeled lactate concentration, from 0.83 ± 0.10 to 0.57 ± 0.14 (*P* = 0.04; *n* = 4; Fig. 4O), and a decrease of 35% in the labeled Glx concentration, from 1.25 ± 0.15 to 0.81 ± 0.09 mmol/L (*P* = 0.023; *n* = 4; Fig. 4P). Although there was a trend toward decreased tumor glucose concentrations in all four tumor models posttreatment, this did not reach statistical significance (Supplementary Fig. S8A–S8H). There were no significant differences in pretreatment tumor volumes between the tumor subtypes (*P* = 0.27) and no significant changes in tumor volumes 24 hours after the completion of chemoradiation (Supplementary Fig. S9A–S9C).

In summary, following the current standard-of-care treatment, there was a reduction in the concentration of labeled lactate in both the glycolytic and mitochondrial subtypes. The decrease in the labeled lactate concentration was greater in glycolytic subtypes than in mitochondrial subtypes. There was no significant difference in the labeled Glx concentration following treatment in the glycolytic subtypes; however, there was a significant decrease in the labeled Glx concentration in the two mitochondrial subtypes.

**Figure 5.**
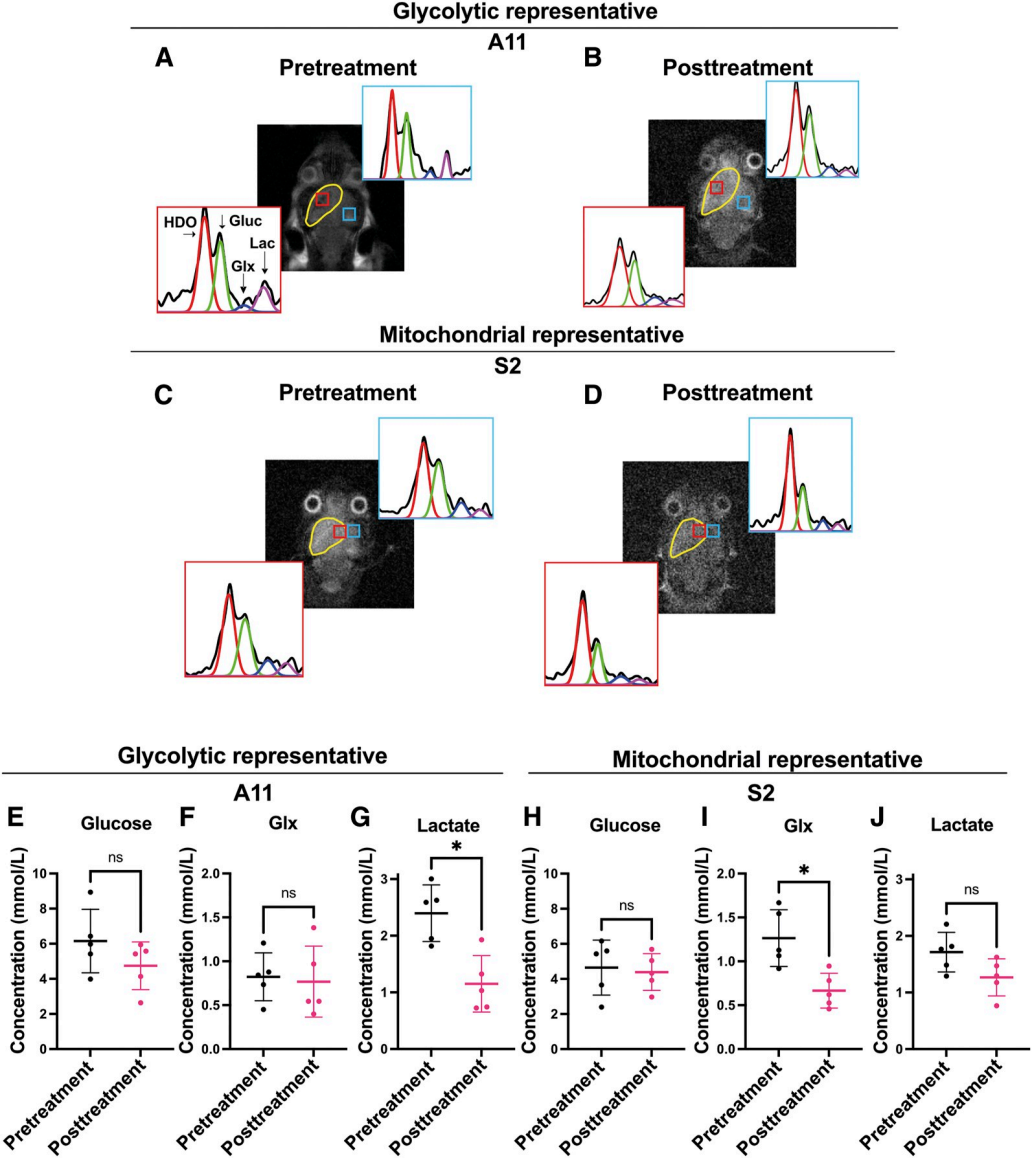
Imaging the effects of chemoradiation on glucose metabolism in tumor models representative of the glycolytic and mitochondrial subtypes. **A-D,** Representative spectra from voxels within the tumor and normal-appearing brain in 3D ^2^H chemical shift images of A11 (**A** and **B**) and S2 (**C** and **D**) tumor–bearing animals infused with 2 g/kg [6,6-^2^H_2_]glucose before (**A** and **C**) and after (**B** and **D**) treatment. Fits to the spectra are shown in color: red, water; green, glucose; blue, Glx; and purple, lactate. The tumor is outlined in yellow on the T_2_-weighted ^1^H image. The locations of the CSI voxels are indicated by the boxes. The spectra are the sum of six 10-minute spectra acquired from 3 × 3 × 9 mm voxels. Labeled glucose, Glx, and lactate concentrations were determined from summed spectra. **E-J,** Labeled glucose (**E**), Glx (**F**), and lactate (**G**) concentrations in A11 tumors before and after treatment, and labeled glucose (**H**), Glx (**I**), and lactate (**J**) concentrations in S2 tumors before and after treatment. ns, not significant; *, *P* < 0.05.

In the cohort of animals used in the experiment shown in Fig. 3, we acquired 3D ^2^H CSI of [6,6′-^2^H_2_]glucose metabolism posttreatment (Fig. 5A–D). There was no significant change in the glucose concentration (Fig. 5E) or ^2^H-labeled Glx concentration (0.82 ± 0.27 mmol/L vs. 0.77 ± 0.40 mmol/L; *P* = 0.77; Fig. 5F) in the A11 tumors posttreatment, but the concentration of ^2^H-labeled lactate decreased significantly, from 2.40 ± 0.50 to 1.15 ± 0.50 mmol/L (*P* = 0.023; Fig. 5G). In a representative of the mitochondrial subtype, S2, there was no significant change in the glucose concentration posttreatment (Fig. 5E), but the ^2^H-labeled Glx concentration decreased from 1.26 ± 0.32 to 0.67 ± 0.20 mmol/L (*P* = 0.013; Fig. 5I) and the ^2^H-labeled lactate concentration from 1.71 ± 0.35 to 1.27 ± 0.33 mmol/L, although this was not statistically significant (*P* = 0.13; Fig. 5J). These changes in ^2^H-labeled lactate and Glx concentrations in A11 and Glx in S2 tumors posttreatment mirrored those observed by coil-localized spectroscopy.

There were no significant differences in ^2^H-labeled glucose, Glx, and lactate concentrations between the normal-appearing brain and A11 and S2 tumors following treatment (Supplementary Fig. S10A–S10F) nor between the normal-appearing brain of A11 and S2 tumor–bearing mice before and after treatment (Supplementary Fig. S11A–S11F).

### Histologic analysis

**Figure 6.**
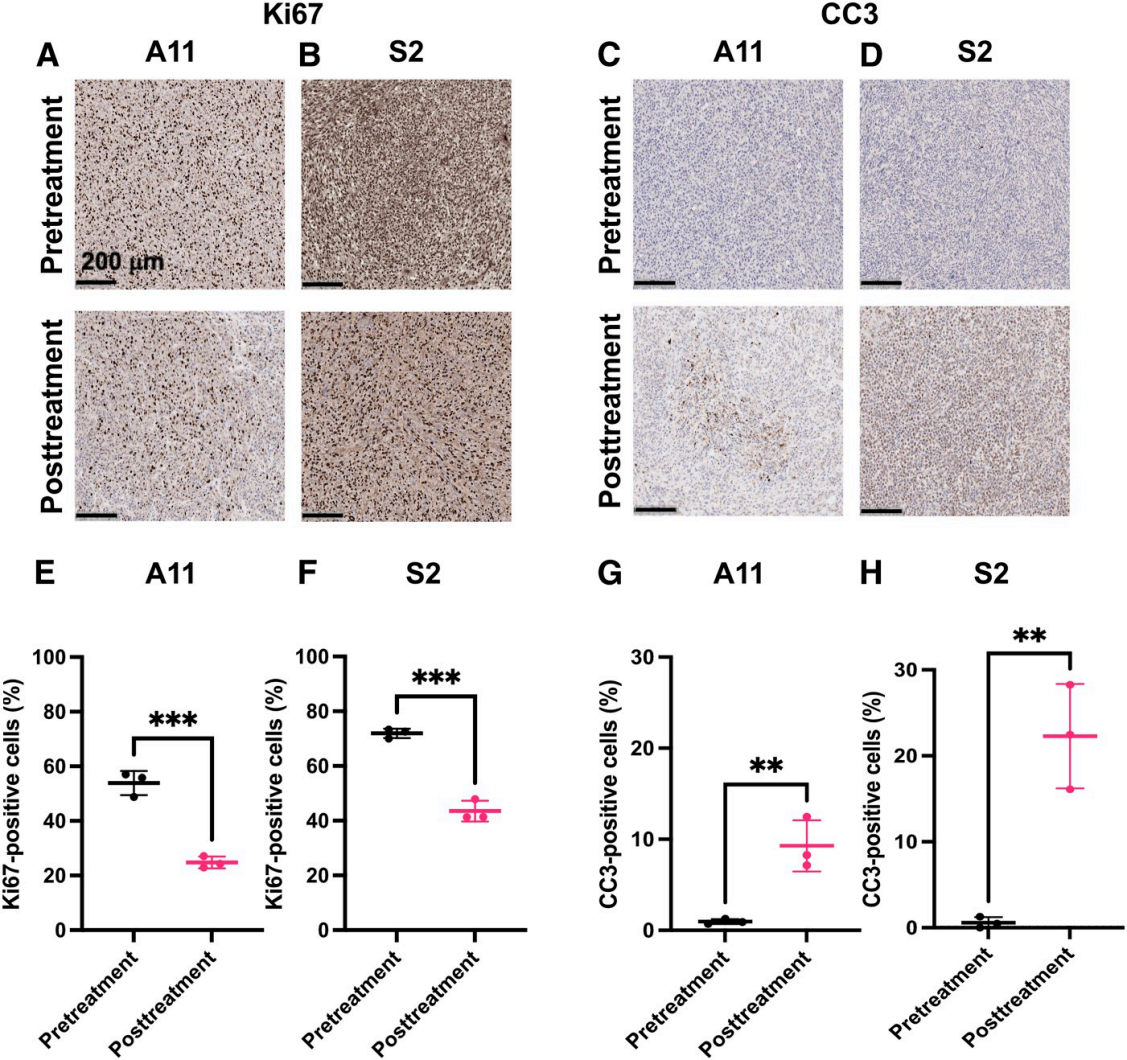
Histologic assessment of tumor cell proliferation and cell death before and 24 hours after treatment. **A-D,** Representative sections of A11 and S2 tumors stained for Ki67 (**A** and **B**) and CC3 (**C** and **D**) pre- and posttreatment with chemoradiation. **E-H,** The percentage of Ki67-positive cells for A11 (**E**) and S2 (**F**) tumors and the percentage of CC3-positive cells for A11 (**G**) and S2 (**H**) tumors before and at 24 hours after completion of treatment. **, *P* < 0.01; ***, *P* < 0.001.

The changes in cell proliferation and cell death following treatment were assessed by staining tumor sections obtained before and 24 hours after chemoradiation for Ki67 and CC3, respectively. There were significant decreases in the percentage of Ki67-positive cells and significant increases in CC3-positive cells in both the A11 and S2 tumors following treatment (Fig. 6). There was a greater increase in cell death in S2 tumors following treatment, in which an increase in CC3-positive cells from 0.59 ± 0.64 to 22.3 ± 6.1% was observed, compared with A11 tumors, in which a smaller increase in CC3-positive cells from 0.95 ± 0.27 to 9.3 ± 2.8% was observed, reflecting an increased sensitivity to chemoradiation. We have demonstrated previously, using an identical treatment protocol to that used here, that S2 tumor–bearing animals show higher levels of tumor cell death and significantly better survival following chemoradiotherapy than mice with A11 and U87 tumors (20).

## Discussion

Metabolic subtypes have been observed in multiple cancer types and are associated with disease prognosis and therapeutic vulnerabilities. In addition to GBM, metabolic subtypes have been identified in other cancers, including pancreatic cancer, in which metabolite profiling revealed glycolytic and lipogenic subtypes. The lipogenic subtype used glucose preferentially in the TCA cycle and for lipid synthesis and had higher oxygen consumption and a greater mitochondrial content, whereas the glycolytic subtype used glucose in aerobic glycolysis (30). These subtypes showed differential sensitivity to inhibitors of glycolysis, glutamine metabolism, and lipid synthesis and were associated with epithelial and mesenchymal subtypes, respectively. In high-grade serous ovarian cancer, a high–oxidative phosphorylation (OXPHOS) subtype was identified, which, like the mitochondrial subtype in GBM, exhibited chronic oxidative stress and increased sensitivity to chemotherapy, whereas a low-OXPHOS subtype displayed a more glycolytic metabolism (31). In diffuse B-cell lymphoma, a subtype was identified that displayed increased mitochondrial energy production and incorporation of nutrient-derived carbons into the TCA cycle, which was selectively sensitive to the inhibition of fatty acid oxidation (32). Similar observations of glycolytic and mitochondrial subtypes have also been observed in esophageal (33) and prostate cancers (34). In summary, classifying tumors based on their metabolic phenotypes may be useful for identifying tumors with better prognosis and their therapeutic vulnerabilities and could potentially be used for treatment selection.

We have shown here that ^2^H imaging of [6,6-^2^H_2_]glucose metabolism can be used to identify, noninvasively, glycolytic and mitochondrial subtypes in orthotopically implanted PDXs of GBM. The metabolic characteristics of the patient-derived cells and the cell line used to generate these GBM models were similar to those reported previously for glycolytic and mitochondrial subtypes of GBM and of other cancer types. The basal OCR and ECAR measurements in cells representative of the mitochondrial (S2 and AT5) and glycolytic subtypes (A11 and U87) and the fold-changes between them were similar to those reported previously for mitochondrial and GPM subtypes of GBM (5). The fold increase in basal oxygen consumption between the glycolytic and mitochondrial subtypes observed here was also similar to that observed between the low-OXPHOS and high-OXPHOS subtypes of high-grade serous ovarian cancer (31) and slightly less than that observed between the non-OXPHOS and OXPHOS subtypes of DBCL (32). The fold increase in labeled lactate production between the glycolytic and mitochondrial subtypes was much less than that reported previously for mitochondrial and GPM subtypes of GBM (5), which is surprising given the similarity in ECAR values; however, it was similar to that observed between the non-OXPHOS and OXPHOS subtypes of DBCL (32).

Following the infusion of [6,6-^2^H_2_]glucose, the mitochondrial subtype xenografts (S2 and AT5) showed higher Glx and lower lactate labeling than the glycolytic subtypes (A11 and U87), indicative of higher TCA cycle and lower glycolytic fluxes, and consistent with the cells having lower ECAR and higher OCR *in vitro*. Localized spectroscopy experiments showed lower tumor lactate concentrations than imaging experiments, presumably reflecting a coil-localized volume that also contained normal brain tissue, in which the lactate concentration was lower. Although we previously fitted the time courses of labeled glucose and lactate to a kinetic model to obtain estimates of glycolytic flux (18) and others have also fitted the time course of Glx labeling to estimate TCA cycle flux (17, 35), we elected here to report the average labeled glucose, lactate, and Glx concentrations following glucose injection because this simpler approach is more likely to be adopted clinically. The measurements of lactate labeling in A11 and S2 tumors were consistent with a previous study in which hyperpolarized [1-^13^C]pyruvate was used to study the metabolism of these tumors (designated GB4 and GB1, respectively, in this earlier study) and also U87 tumors. A11 and U87 tumors showed a hyperpolarized [1-^13^C]lactate/[1-^13^C]pyruvate signal intensity ratio of ∼0.3, compared with a ratio of ∼0.1 in S2 tumors, which was indistinguishable from that in the normal-appearing brain (13). Here, the imaging experiments showed a ^2^H-labeled lactate concentration of 2.4 ± 0.50 mmol/L in A11 tumors compared with 1.7 mmol/L ± 0.35 mmol/L in S2 tumors, which was similar to the concentration in normal-appearing brain tissue. Lactate labeling from injected hyperpolarized [1-^13^C]pyruvate depends on pyruvate delivery, the size of the intracellular lactate pool (which depends on glycolytic activity), and the activities of lactate dehydrogenase (LDH) and monocarboxylate transporters (MCT). The expression of glycolytic enzymes, including LDH, hexokinase II (HK2), and MCTs, is driven by c-Myc (36). In a previous study, we showed that c-Myc, LDH, HK2, and plasma membrane MCT concentrations were greater in A11 cells than in S2 cells and that inducible short hairpin RNA–mediated knockdown of c-Myc expression in A11 cells resulted in decreased LDH and HK2 expression and a decrease in lactate labeling.

Glx labeling in A11 tumors was similar to that in normal-appearing brain tissue but higher in the mitochondrial subtype S2 tumor. Conversely, lactate labeling in S2 tumors was similar to that in normal-appearing brain tissue but higher in the glycolytic subtype A11 tumor. The nearly quantitative reciprocity between lactate and Glx labeling in A11 and S2 tumors implies that in the more oxidative S2 tumor, glucose is diverted away from lactate production and into the TCA cycle. A recent study of five primary solid tumors showed that TCA cycle activity was suppressed in the tumors studied, and although glycolytic flux increased, this could not compensate for the decrease in TCA cycle flux in terms of ATP production (37). The authors suggested that normal tissue functions in tumors are suppressed, leading to a lower demand for ATP, and therefore the rate of ATP production, and that the ATP produced is used to drive tumor cell proliferation. The study used [U-^13^C]lactate to measure TCA cycle flux, and because lactate is not a significant substrate for the brain, TCA cycle flux was not measured in this tissue. However, measurements of TCA cycle flux in two breast cancer models of lung metastasis showed an increased TCA cycle flux in the metastases, but similar glucose use, as determined using [1-^13^C]2-deoxyglucose, to the primary tumors. In these GBM models, we found no evidence of suppression of TCA cycle activity when compared with normal-appearing brain tissue, with a TCA cycle flux that was similar to normal brain tissue in the glycolytic subtype A11 tumor and increased TCA cycle flux in the mitochondrial subtype S2 tumor. This may be explained by the suppression of TCA cycle flux in normal brain tissue by the isoflurane anesthesia used here (17). However, significant tumor TCA cycle activity has been observed in patients with GBM, in which their infusion with [U-^13^C]glucose and ^13^C NMR analysis of tumor extracts demonstrated substantial incorporation of glucose carbon into TCA cycle intermediates (38).

The response of patients with GBM to standard-of-care chemoradiation treatment is conventionally assessed using contrast-enhanced MRI, in which a decrease in the size of the enhancing tumor is used as an indication of treatment response (39). However, this technique reflects only the integrity of the blood–brain barrier, does not provide any information on tumor cell activity, and can be confounded by the phenomenon of pseudoprogression, in which contrast enhancement can occur without true tumor growth (40). ^1^H MRS measurements of tumor cell metabolic activity have been shown to be superior to more advanced MRI measurements, including diffusion-weighted and perfusion-based MRI measurements, in distinguishing progression from pseudoprogression (41). Using a model in which GBM cells were implanted in irradiated brain tissue, DMI measurements of [6,6-^2^H_2_]glucose metabolism showed a higher lactate/Glx ratio in the tumor than in regions of radiation necrosis, which showed a ratio more similar to that in the normal brain (42). DMI measurements with [^2^H_9_]choline, which showed high uptake in a GBM model when compared with the normal brain, have also been proposed as a method for distinguishing progression from pseudoprogression (43). We showed previously that ^13^C MRI measurements of hyperpolarized [1-^13^C]pyruvate metabolism can be used to detect early responses to chemoradiation. In A11 tumors, there was an approximately 29% decrease in lactate labeling at 72 hours posttreatment (13), which is much less than the decrease in ^2^H-labeled lactate concentration determined here at 24 hours posttreatment, which decreased from 2.40 ± 0.50 to 1.15 ± 0.50 mmol/L. There was no significant change in the labeled Glx concentration. The sensitivity for detecting response was lower in a representative of the mitochondrial subtype, S2, in which the ^2^H-labeled lactate concentration declined from 1.71 ± 0.35 to 1.27 ± 0.33 mmol/L posttreatment. Glx labeling, which was higher in the mitochondrial subtypes, showed a larger decrease in labeling posttreatment, from 1.3 ± 0.32 to 0.67 ± 0.20 mmol/L, in S2 tumors but no decrease in the glycolytic subtype A11, in which it remained at ∼0.8 mmol/L. Some of the decreases in lactate and Glx labeling may be explained by the decrease in [6,6-^2^H_2_]glucose concentrations in the tumors posttreatment. Although flux into the TCA cycle has been measured in a glioma tumor model using hyperpolarized [1-^13^C]pyruvate, from measurements of labeled bicarbonate (44), the signal from bicarbonate was too small to detect treatment response in our previous study (13). In summary, measurements of ^2^H-labeled lactate in glycolytic subtype tumors and ^2^H-labeled Glx in mitochondrial subtypes can be used to detect the response to chemoradiation, and this seems to be more sensitive than detecting the response using hyperpolarized [1-^13^C]pyruvate. However, this is a much less sensitive approach for detecting treatment-related cell death than ^2^H imaging of [2,3-^2^H_2_]fumarate metabolism, in which at 48 hours postchemoradiation, we observed a 340% increase in the [2,3-^2^H_2_]malate/[2,3-^2^H_2_]fumarate ratio in A11 tumors and a 755% increase in S2 tumors (20).

DMI of [6,6′-^2^H_2_]glucose metabolism has already been used in patients with GBM, in which higher lactate labeling and lower Glx labeling were observed in the tumor than in the normal brain following oral administration of the labeled glucose (23). We have shown here that this technique has the potential to be used clinically to differentiate between the glycolytic and mitochondrial subtypes of GBM. This could be used to indicate disease prognosis, to select treatments that target the metabolic vulnerabilities of these subtypes, and in follow-up studies to detect very early responses to treatment, thus validating treatment selection.

## Supplementary Material

Supplementary Data Supplementary Methods and Supplementary Figures S1–S11

## References

[1] Vander Heiden MG , Cantley LC, Thompson CB. Understanding the Warburg effect: the metabolic requirements of cell proliferation. Science 2009;324:1029–33. 19460998 10.1126/science.1160809 PMC2849637

[2] Hanahan D , Weinberg RA. Hallmarks of cancer: the next generation. Cell 2011;144:646–74. 21376230 10.1016/j.cell.2011.02.013

[3] Hoxhaj G , Manning BD. The PI3K-AKT network at the interface of oncogenic signalling and cancer metabolism. Nat Rev Cancer 2020;20:74–88. 31686003 10.1038/s41568-019-0216-7 PMC7314312

[4] Faubert B , Solmonson A, DeBerardinis RJ. Metabolic reprogramming and cancer progression. Science 2020;368:eaaw5473. 32273439 10.1126/science.aaw5473 PMC7227780

[5] Garofano L , Migliozzi S, Oh YT, D’Angelo F, Najac RD, Ko A, . Pathway-based classification of glioblastoma uncovers a mitochondrial subtype with therapeutic vulnerabilities. Nat Cancer 2021;2:141–56. 33681822 10.1038/s43018-020-00159-4 PMC7935068

[6] Wang Q , Hu B, Hu X, Kim H, Squatrito M, Scarpace L, . Tumor evolution of glioma-intrinsic gene expression subtypes associates with immunological changes in the microenvironment. Cancer Cell 2017;32:42–56.e6. 28697342 10.1016/j.ccell.2017.06.003 PMC5599156

[7] Lasorella A , Iavarone A. The making of the glioblastoma classification. Br J Cancer 2021;125:4–6. 33767415 10.1038/s41416-021-01360-7 PMC8257735

[8] Brindle K . New approaches for imaging tumour responses to treatment. Nat Rev Cancer 2008;8:94–107. 18202697 10.1038/nrc2289

[9] Kernstine KH , Faubert B, Do QN, Rogers TJ, Hensley CT, Cai L, . Does tumor FDG-PET avidity represent enhanced glycolytic metabolism in non-small cell lung cancer? Ann Thorac Surg 2020;109:1019–25. 31846640 10.1016/j.athoracsur.2019.10.061 PMC7370816

[10] Galldiks N , Niyazi M, Grosu AL, Kocher M, Langen KJ, Law I, . Contribution of PET imaging to radiotherapy planning and monitoring in glioma patients—a report of the PET/RANO group. Neuro Oncol 2021;23:881–93. 33538838 10.1093/neuonc/noab013 PMC8168815

[11] Parent EE , Johnson DR, Gleason T, Villanueva-Meyer JE. Neuro-Oncology Practice Clinical Debate: FDG PET to differentiate glioblastoma recurrence from treatment-related changes. Neurooncol Pract 2021;8:518–25. 34594566 10.1093/nop/npab027 PMC8475205

[12] Nichelli L , Casagranda S. Current emerging MRI tools for radionecrosis and pseudoprogression diagnosis. Curr Opin Oncol 2021;33:597–607. 34534142 10.1097/CCO.0000000000000793 PMC8528135

[13] Mair R , Wright AJ, Ros S, Hu DE, Booth T, Kreis F, . Metabolic imaging detects low levels of glycolytic activity that vary with levels of c-myc expression in patient-derived xenograft models of glioblastoma. Cancer Res 2018;78:5408–18. 30054337 10.1158/0008-5472.CAN-18-0759

[14] Miloushev VZ , Granlund KL, Boltyanskiy R, Lyashchenko SK, DeAngelis LM, Mellinghoff IK, . Metabolic imaging of the human brain with hyperpolarized ^13^C pyruvate demonstrates ^13^C lactate production in brain tumor patients. Cancer Res 2018;78:3755–60. 29769199 10.1158/0008-5472.CAN-18-0221 PMC6050093

[15] Zaccagna F , McLean MA, Grist JT, Kaggie J, Mair R, Riemer F, . Imaging glioblastoma metabolism by using hyperpolarized [1-^13^C]pyruvate demonstrates heterogeneity in lactate labeling: a proof of principle study. Radiol Imaging Cancer 2022;4:e210076. 35838532 10.1148/rycan.210076 PMC9360994

[16] Kurhanewicz J , Vigneron DB, Ardenkjaer-Larsen JH, Bankson JA, Brindle K, Cunningham CH, . Hyperpolarized ^13^C MRI: path to clinical translation in oncology. Neoplasia 2019;21:1–16. 30472500 10.1016/j.neo.2018.09.006 PMC6260457

[17] Lu M , Zhu XH, Zhang Y, Mateescu G, Chen W. Quantitative assessment of brain glucose metabolic rates using *in vivo* deuterium magnetic resonance spectroscopy. J Cereb Blood Flow Metab 2017;37:3518–30. 28503999 10.1177/0271678X17706444 PMC5669347

[18] Kreis F , Wright AJ, Hesse F, Fala M, Hu DE, Brindle KM. Measuring tumor glycolytic flux in vivo by using fast deuterium MRI. Radiology 2020;294:289–96. 31821119 10.1148/radiol.2019191242

[19] Hesse F , Somai V, Kreis F, Bulat F, Wright AJ, Brindle KM. Monitoring tumor cell death in murine tumor models using deuterium magnetic resonance spectroscopy and spectroscopic imaging. Proc Natl Acad Sci U S A 2021;118:e2014631118. 33727417 10.1073/pnas.2014631118 PMC8000230

[20] Hesse F , Wright AJ, Somai V, Bulat F, Kreis F, Brindle KM. Imaging glioblastoma response to radiotherapy using ^2^H magnetic resonance spectroscopy measurements of fumarate metabolism. Cancer Res 2022;82:3622–33. 35972377 10.1158/0008-5472.CAN-22-0101 PMC9530651

[21] De Feyter HM , de Graaf RA. Deuterium metabolic imaging—back to the future. J Magn Reson 2021;326:106932. 33902815 10.1016/j.jmr.2021.106932 PMC8083995

[22] Low CML , Wright AJ, Hesse F, Cao J, Brindle KM. Metabolic imaging with deuterium labeled substrates. Prog Nucl Magn Reson Spectrosc 2023;134–5:39–51. 10.1016/j.pnmrs.2023.02.002 37321757

[23] De Feyter HM , Behar KL, Corbin ZA, Fulbright RK, Brown PB, McIntyre S, . Deuterium metabolic imaging (DMI) for MRI-based 3D mapping of metabolism *in vivo*. Sci Adv 2018;4:eaat7314. 30140744 10.1126/sciadv.aat7314 PMC6105304

[24] Ruhm L , Avdievich N, Ziegs T, Nagel AM, De Feyter HM, de Graaf RA, . Deuterium metabolic imaging in the human brain at 9.4 Tesla with high spatial and temporal resolution. Neuroimage 2021;244:118639. 34637905 10.1016/j.neuroimage.2021.118639 PMC8591372

[25] Gordon JW , Chen HY, Autry A, Park I, Van Criekinge M, Mammoli D, . Translation of Carbon-13 EPI for hyperpolarized MR molecular imaging of prostate and brain cancer patients. Magn Reson Med 2019;81:2702–9. 30375043 10.1002/mrm.27549 PMC6372313

[26] McAbee JH , Degorre-Kerbaul C, Valdez K, Wendler A, Shankavaram UT, Watts C, . Detection of glioblastoma intratumor heterogeneity in radiosensitivity using patient-derived neurosphere cultures. J Neurooncol 2020;149:383–90. 33057920 10.1007/s11060-020-03643-0 PMC8942292

[27] Bligh EG , Dyer WJ. A rapid method of total lipid extraction and purification. Can J Biochem Physiol 1959;37:911–7. 13671378 10.1139/o59-099

[28] Hesse F , Wright A, Bulat F, Kreis F, Brindle KM. Assessment of the sensitivity of ^2^H MR spectroscopy measurements of [2,3-^2^H_2_]fumarate metabolism for detecting tumor cell death. NMR Biomed 2023;36:e4965. 37148156 10.1002/nbm.4965 PMC10909471

[29] Fala M , Ros S, Sawle A, Rao JU, Tsyben A, Tronci L, . The role of branched-chain aminotransferase 1 in driving glioblastoma cell proliferation and invasion varies with tumor subtype. Neurooncol Adv 2023;5:vdad120. 37885806 10.1093/noajnl/vdad120 PMC10599397

[30] Daemen A , Peterson D, Sahu N, McCord R, Du X, Liu B, . Metabolite profiling stratifies pancreatic ductal adenocarcinomas into subtypes with distinct sensitivities to metabolic inhibitors. Proc Natl Acad Sci U S A 2015;112:E4410-7. 26216984 10.1073/pnas.1501605112 PMC4538616

[31] Gentric G , Kieffer Y, Mieulet V, Goundiam O, Bonneau C, Nemati F, . PML-regulated mitochondrial metabolism enhances chemosensitivity in human ovarian cancers. Cell Metab 2019;29:156–73.e10. 30244973 10.1016/j.cmet.2018.09.002 PMC6331342

[32] Caro P , Kishan AU, Norberg E, Stanley IA, Chapuy B, Ficarro SB, . Metabolic signatures uncover distinct targets in molecular subsets of diffuse large B cell lymphoma. Cancer Cell 2012;22:547–60. 23079663 10.1016/j.ccr.2012.08.014 PMC3479446

[33] King RJ , Qiu F, Yu F, Singh PK. Metabolic and immunological subtypes of esophageal cancer reveal potential therapeutic opportunities. Front Cell Dev Biol 2021;9:667852. 34307352 10.3389/fcell.2021.667852 PMC8295652

[34] Mossa F , Robesti D, Sumankalai R, Corey E, Titus M, Kang Y, . Subtype and site specific-induced metabolic vulnerabilities in prostate cancer. Mol Cancer Res 2023;21:51–61. 36112348 10.1158/1541-7786.MCR-22-0250 PMC9812897

[35] Simões RV , Henriques RN, Cardoso BM, Fernandes FF, Carvalho T, Shemesh N. Glucose fluxes in glycolytic and oxidative pathways detected *in vivo* by deuterium magnetic resonance spectroscopy reflect proliferation in mouse glioblastoma. Neuroimage Clin 2022;33:102932. 35026626 10.1016/j.nicl.2021.102932 PMC8760481

[36] Stine ZE , Walton ZE, Altman BJ, Hsieh AL, Dang CV. MYC, metabolism, and cancer. Cancer Discov 2015;5:1024–39. 26382145 10.1158/2159-8290.CD-15-0507 PMC4592441

[37] Bartman CR , Weilandt DR, Shen Y, Lee WD, Han Y, TeSlaa T, . Slow TCA flux and ATP production in primary solid tumours but not metastases. Nature 2023;614:349–57. 36725930 10.1038/s41586-022-05661-6 PMC10288502

[38] Maher EA , Marin-Valencia I, Bachoo RM, Mashimo T, Raisanen J, Hatanpaa KJ, . Metabolism of [U-^13^C]glucose in human brain tumors in vivo. NMR Biomed 2012;25:1234–44. 22419606 10.1002/nbm.2794 PMC3406255

[39] Wen PY , Macdonald DR, Reardon DA, Cloughesy TF, Sorensen AG, Galanis E, . Updated response assessment criteria for high-grade gliomas: response assessment in neuro-oncology working group. J Clin Oncol 2010;28:1963–72. 20231676 10.1200/JCO.2009.26.3541

[40] Thust SC , van den Bent MJ, Smits M. Pseudoprogression of brain tumors. J Magn Reson Imaging 2018;48:571–89. 29734497 10.1002/jmri.26171 PMC6175399

[41] van Dijken BRJ , van Laar PJ, Holtman GA, van der Hoorn A. Diagnostic accuracy of magnetic resonance imaging techniques for treatment response evaluation in patients with high-grade glioma, a systematic review and meta-analysis. Eur Radiol 2017;27:4129–44. 28332014 10.1007/s00330-017-4789-9 PMC5579204

[42] Ge X , Song KH, Engelbach JA, Yuan L, Gao F, Dahiya S, . Distinguishing tumor admixed in a radiation necrosis (RN) background: ^1^H and ^2^H MR with a novel mouse brain-tumor/RN model. Front Oncol 2022;12:885480. 35712497 10.3389/fonc.2022.885480 PMC9196939

[43] Ip KL , Thomas MA, Behar KL, de Graaf RA, De Feyter HM. Mapping of exogenous choline uptake and metabolism in rat glioblastoma using deuterium metabolic imaging (DMI). Front Cell Neurosci 2023;17:1130816. 37187610 10.3389/fncel.2023.1130816 PMC10175635

[44] Park JM , Recht LD, Josan S, Merchant M, Jang T, Yen YF, . Metabolic response of glioma to dichloroacetate measured *in vivo* by hyperpolarized ^13^C magnetic resonance spectroscopic imaging. Neuro Oncol 2013;15:433–41. 23328814 10.1093/neuonc/nos319 PMC3607261

