# Supplementary material for: Deuterium Metabolic Imaging Differentiates Glioblastoma Metabolic Subtypes and Detects Early Response to Chemoradiotherapy
Source: Cancer Res. 2024 Apr 18;84(12):1996–2008. doi: 10.1158/0008-5472.CAN-23-2552 (PMC11176915; doi:10.1158/0008-5472.CAN-23-2552)
Supplement: Supplementary Data — Supplementary Methods and Supplementary Figures S1–S11 [file can-23-2552_supplementary_data_suppsd.docx]

**SUPPLEMENTARY INFORMATION**

Deuterium metabolic imaging differentiates glioblastoma metabolic subtypes and detects their early response to chemoradiotherapy

Jacob Chen Ming Low, Jianbo Cao, Friederike Hesse, Alan J. Wright†, Anastasia Tsyben, Islam Alshamleh, Richard Mair and Kevin M. Brindle

Cancer Research UK Cambridge Institute, University of Cambridge, Li Ka Shing Centre, Robinson Way, Cambridge, CB2 0RE, UK

†Present address: Guy’s and St Thomas’s NHS Foundation Trust, St Thomas’ Hospital, Westminster Bridge Road, London SE1 7EH

**Supplementary methods**

**Classification of tumor subtype using RNA sequencing data**

Garofano et al. (1) described a transcriptomic approach to classify glioblastomas into four subtypes; Glycolytic (GPM), Mitochondrial (MTC), Neuronal (NEU) and Proliferative (PPR). We applied this classification to A11 and S2 xenografts implanted orthotopically in rats using a previously published RNA sequencing dataset (2). Quantitation of gene expression, data processing, normalization and annotation are described in this previous publication. The A11 and S2 xenografts were classified according to TPM normalized counts for the signature genes in each subtype. Briefly, Garofano et al. (1) listed the top 50 enriched genes that characterized each glioblastoma subtype. The classification algorithm calculates a probability score for each subtype, which is an estimate of whether the expression of genes within a gene set is higher than that of genes outside this gene set. A positive score means enrichment while a negative score means down regulation. Analysis was performed in R (version R 4.3.1; RRID:SXCR_001905).

**Supplementary figures**

**
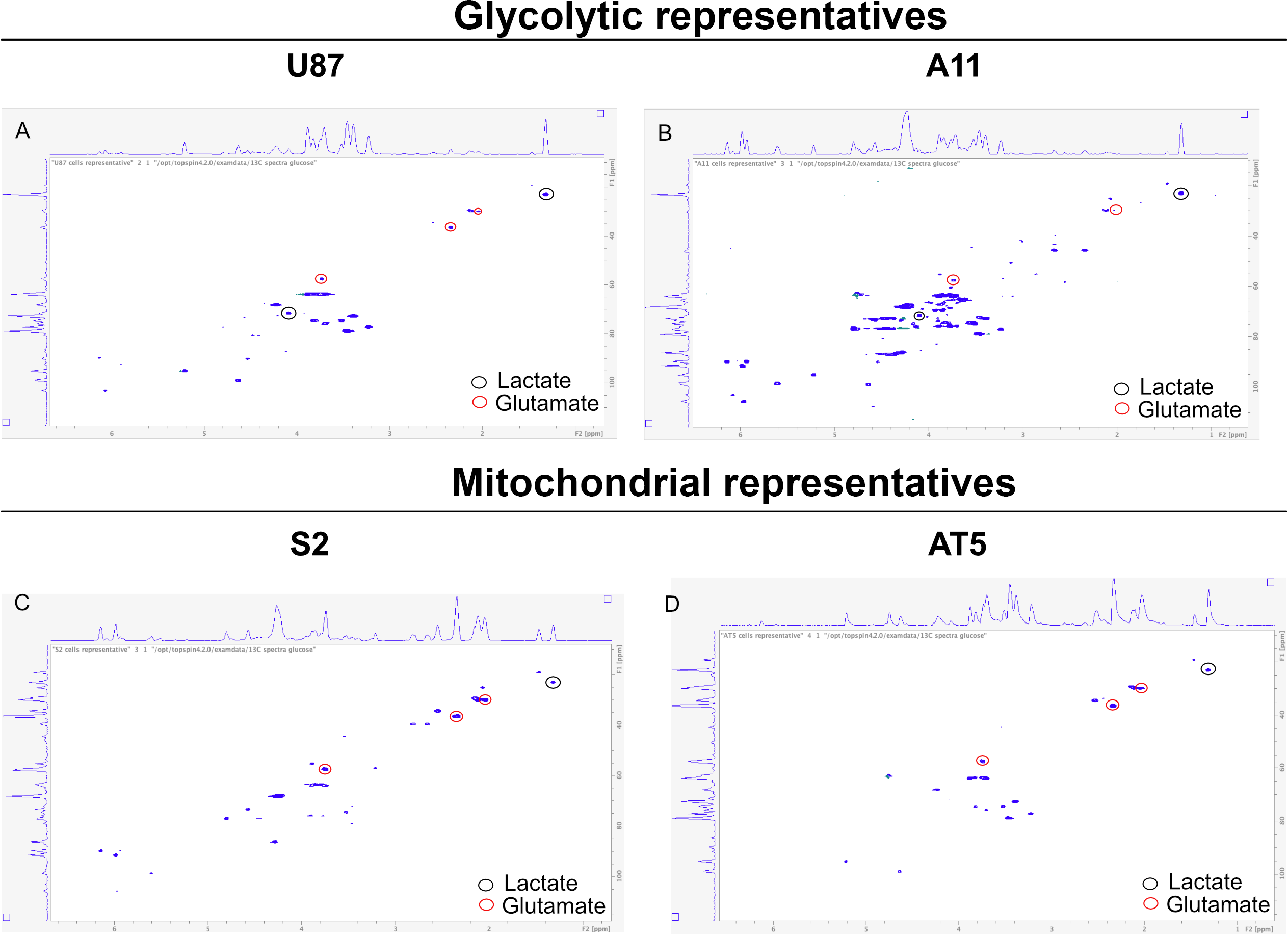
**

**Supplementary Figure S1. Representative ^1^H - ^13^C Heteronuclear Single Quantum Coherence (HSQC) spectra of extracts of U87, A11, S2 and AT5 cells that had been incubated with 10 mM [U-^13^C]glucose.**

Lactate peaks are circled in black and glutamate peaks in red.

**
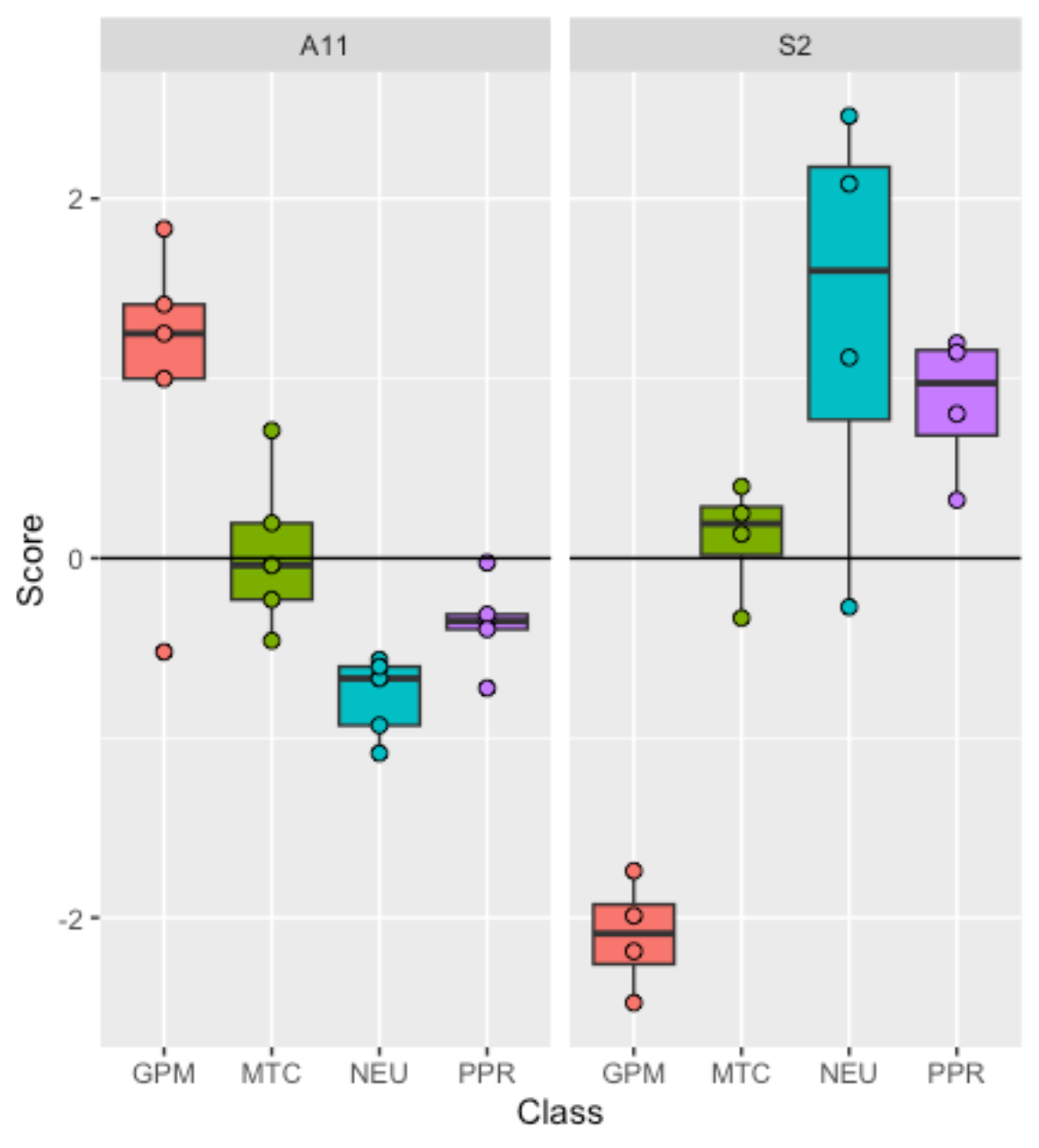
**

**Supplementary Figure 2. RNA sequencing-based classification of A11 and S2 xenografts**

Each point represents a biological replicate. Upregulation in the expression levels of the glycolytic gene signature was observed in A11 tumors, whereas S2 tumors showed upregulation in expression of the mitochondrial gene signature and downregulation in the expression of the glycolytic gene signature. Abbreviations: GPM, glycolytic; MTC, mitochondrial; NEU, neuronal; PPR, proliferative/progenitor.


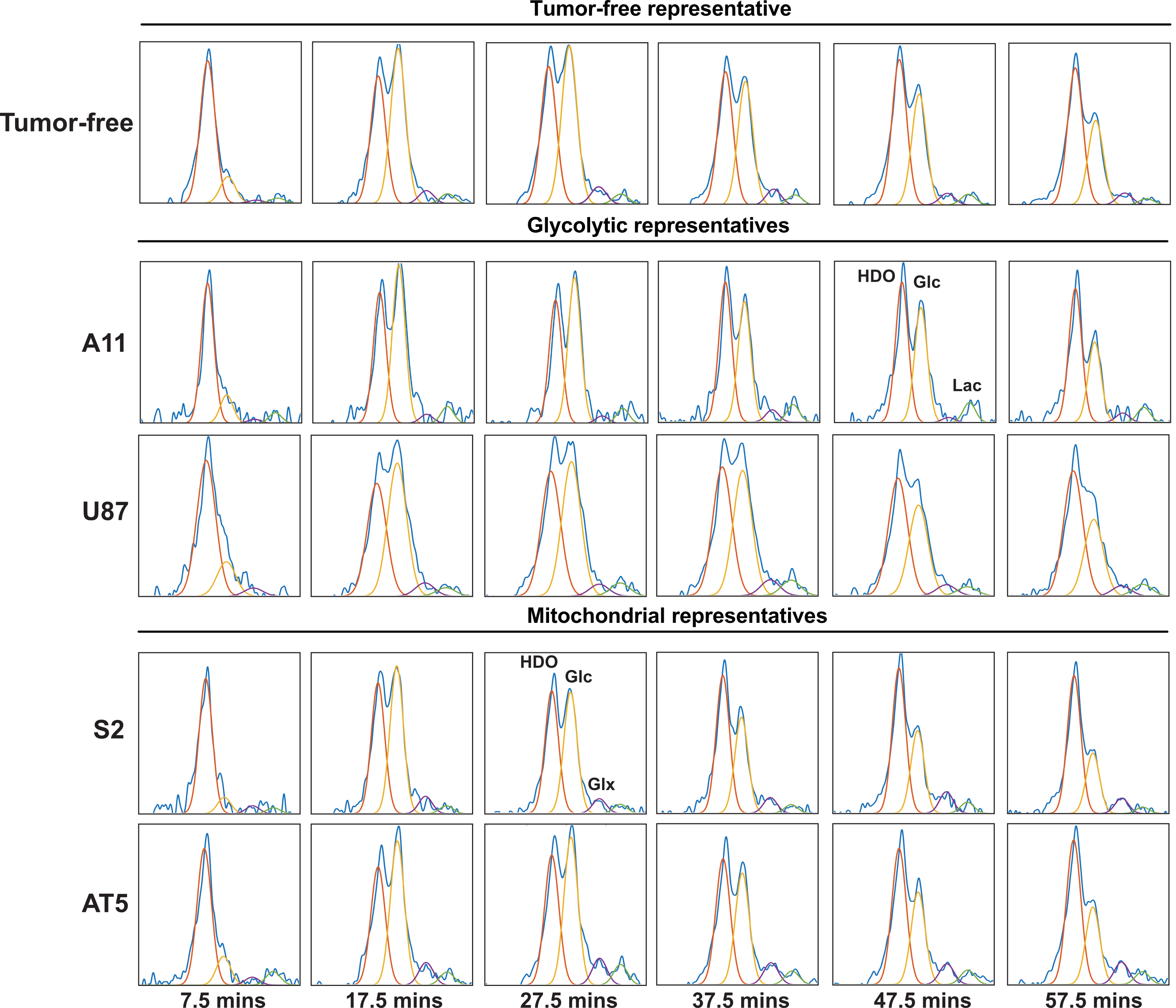


**Supplementary Figure 3. Representative coil-localized ^2^H spectra from tumor-free mice and from animals implanted with the four tumor models**

Serial 5 min tumor ^2^H spectra were acquired from the two glycolytic (A11 and U87) (A-H) and two mitochondrial subtype tumors (S2 and AT5) following intravenous injection of 2g/kg [6,6’-^2^H_2_]glucose. The mid-point times of spectrum acquisition are shown. The spectra are shown in blue and the fits are shown for labeled water (HDO) in red, glucose (Glc) in yellow, glutamine/glutamate (Glx) in purple, and lactate (Lac) in green.


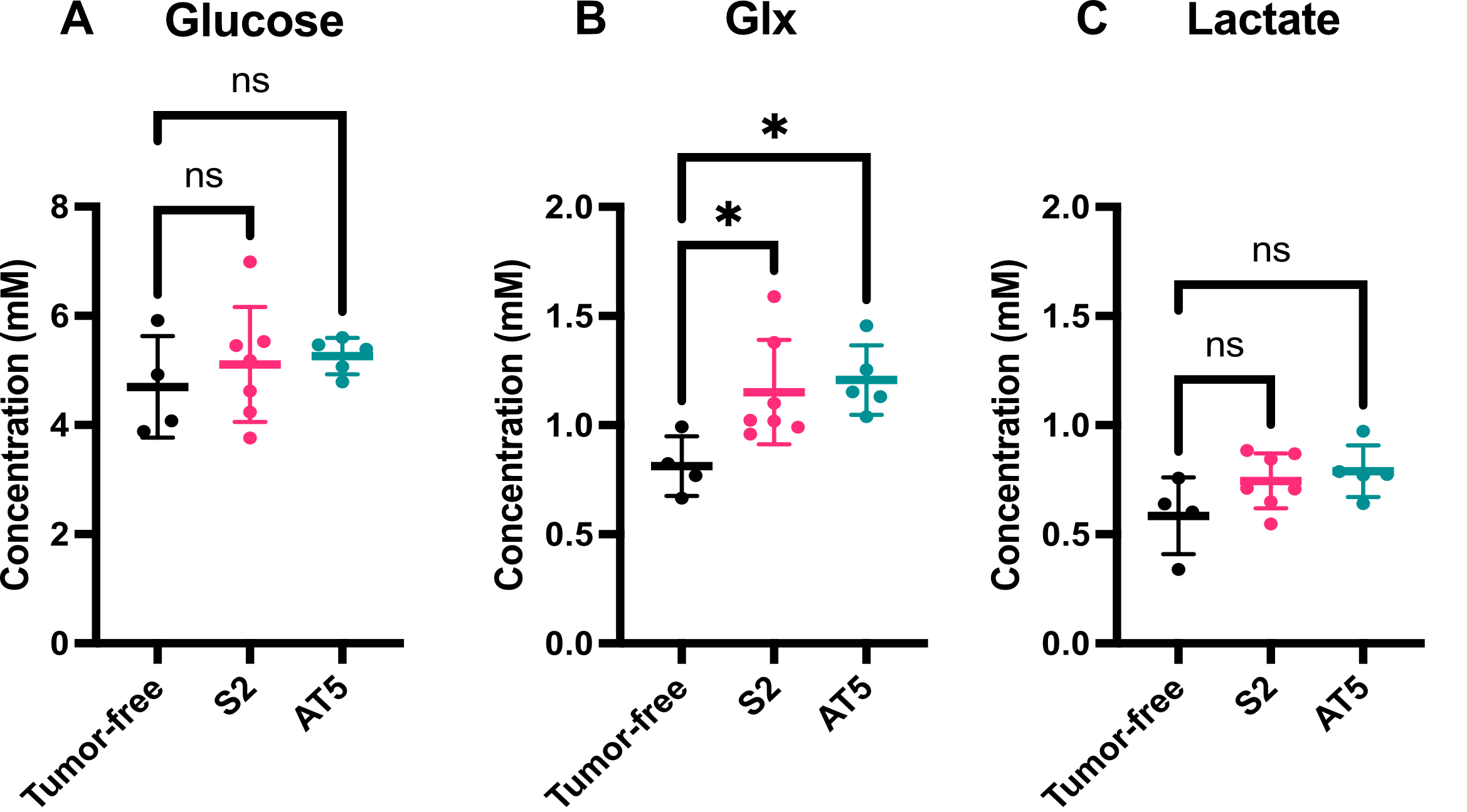


**Supplementary Figure 4. Coil-localized ^2^H MRS measurements of labeled glucose, Glx and lactate in mitochondrial subtype tumors and in the brains of tumor-free animals.**

Concentrations of ^2^H-labeled glucose (A), Glx (B), and lactate (C) measured between 20 minutes and 65 minutes following injection of 2g/kg [6,6’-^2^H_2_]glucose in the brains of tumor-free mice and mice implanted with the two mitochondrial subtype tumors. A one-way ANOVA showed that there were no significant differences in the labeled glucose or lactate concentrations between the normal brain and the mitochondrial subtypes. A *post hoc* Dunnett’s multiple comparison analysis showed significant differences in the concentrations of labeled Glx between normal brain and the mitochondrial subtype tumors. ns, not significant; *p <0.05.

**
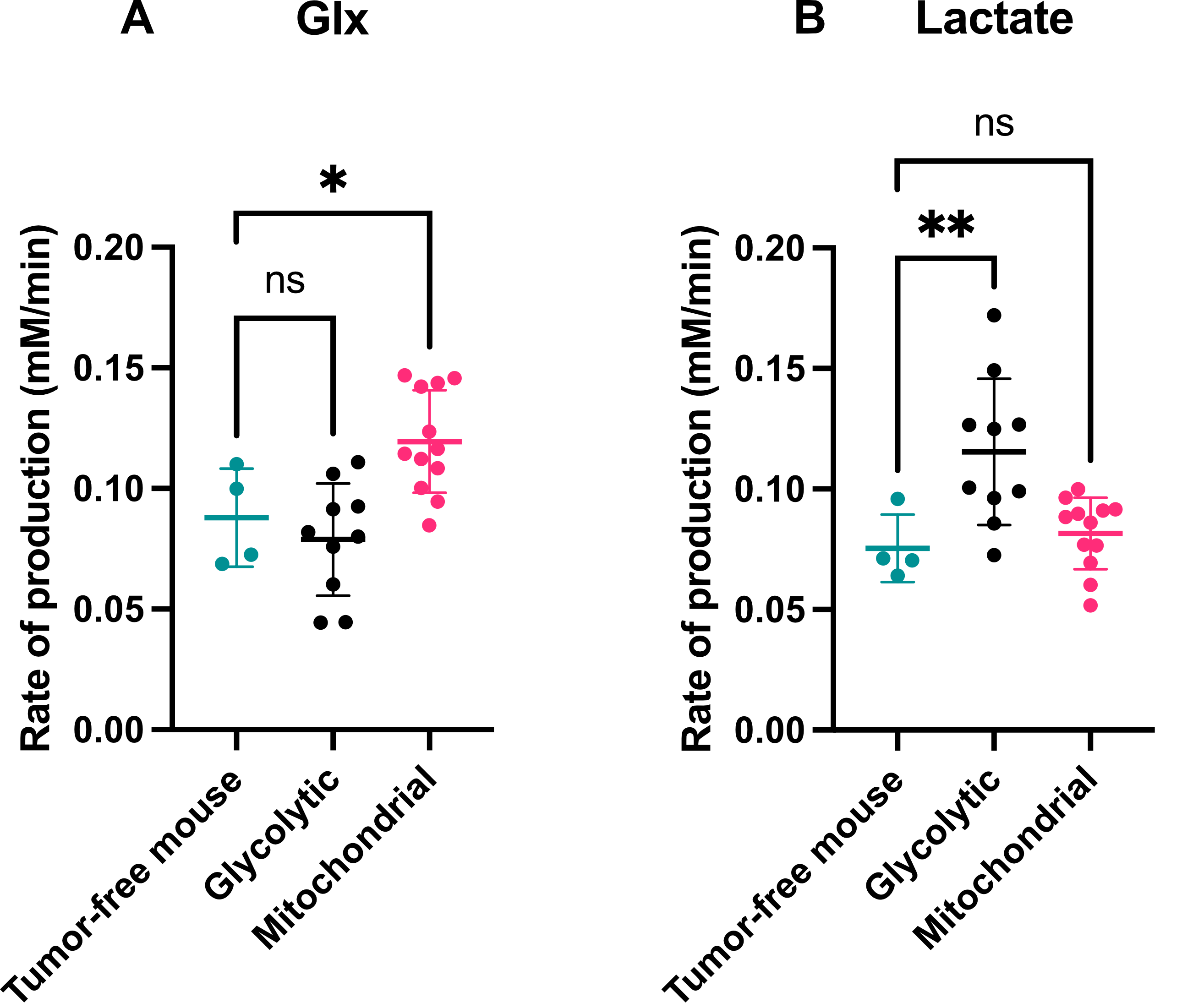
**

**Supplementary Figure 5. Initial rates of ^2^H-labeled Glx (A) and lactate (B) production measured using coil-localized ^2^H MRS in the first 20 min following injection of 2g/kg [6,6-^2^H_2_]glucose in tumor-free animals and in animals implanted with glycolytic and mitochondrial subtype tumors.**

The mitochondrial tumor subtype tumors (S2, AT5) showed significantly higher rates of labeled Glx production, but similar rates of labeled lactate production, compared to the tumor-free animals. The glycolytic tumor subtypes (A11, U87) showed significantly higher rates of labeled lactate production, but similar rates of labeled Glx production, compared to the tumor-free animals. ns, not significant; *p <0.05; **p<0.01.


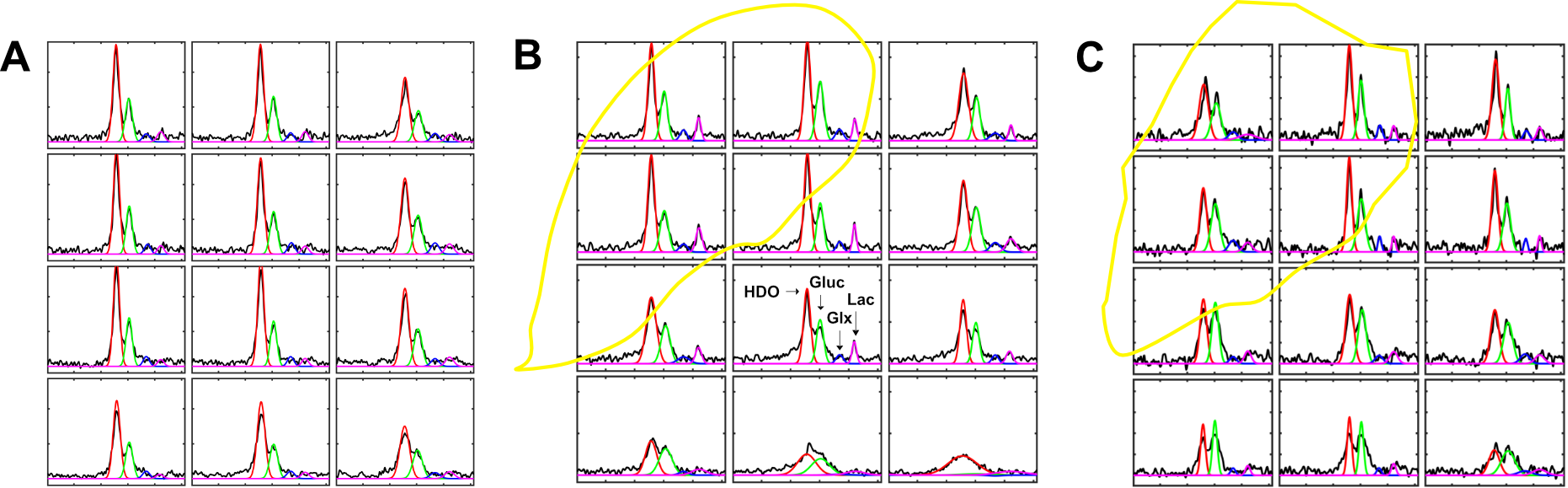


**Supplementary Figure 6. Spectra from the 12 voxels in the 3D chemical shift images covering the brain of a representative tumor-free animal (A), A11 tumor-bearing mouse (B) and S2 tumor-bearing mouse (C).**

The location of the tumor is highlighted in yellow in (B) & (C). The spectra are shown in black and the fits are shown for labeled water (HDO) in red, glucose (Glc) in green, glutamine/glutamate (Glx) in blue, and lactate (Lac) in purple.


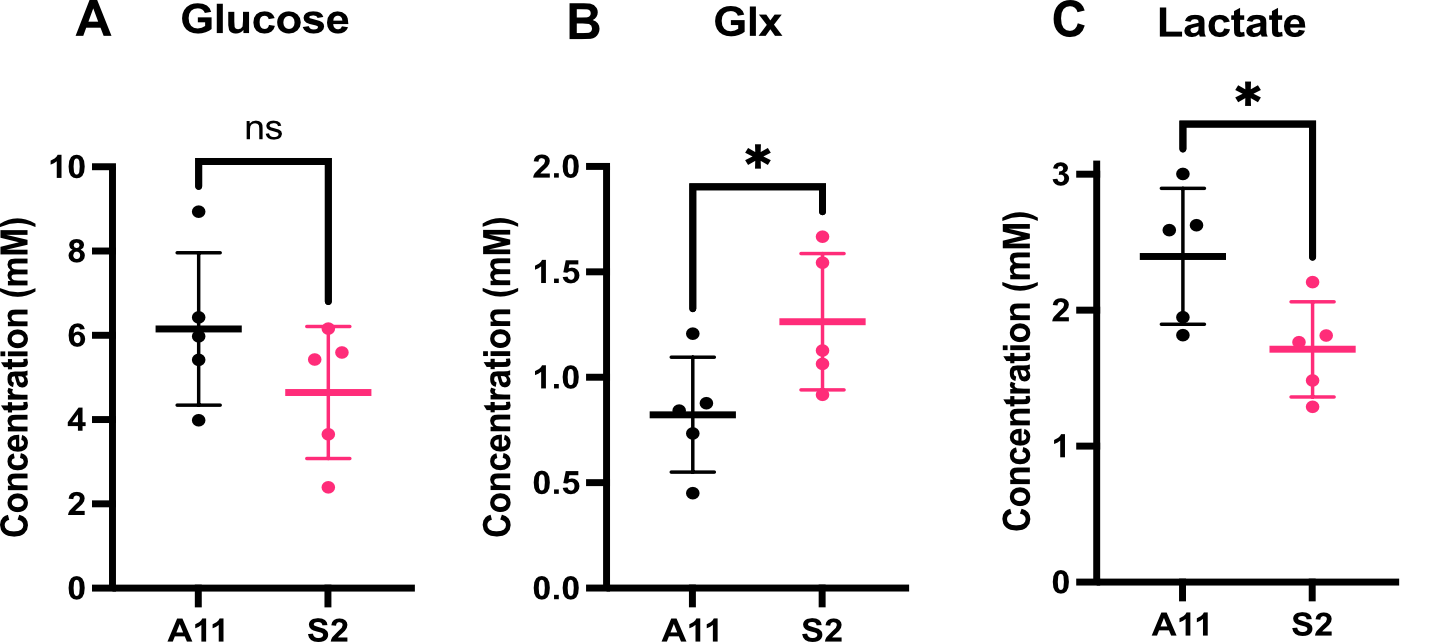


**Supplementary Figure 7. 3D ^2^H CSI of [6,6-^2^H_2_]glucose metabolism in A11 and S2 tumors.**

Comparison of labeled glucose (A), Glx (B), and lactate (C) concentrations measured from 10-minute spectra acquired over a period of 60 min, starting 10 min following injection of 2 g/kg [6,6-^2^H_2_]glucose in A11 and S2 tumor-bearing mice. ns, not significant; *p <0.05.


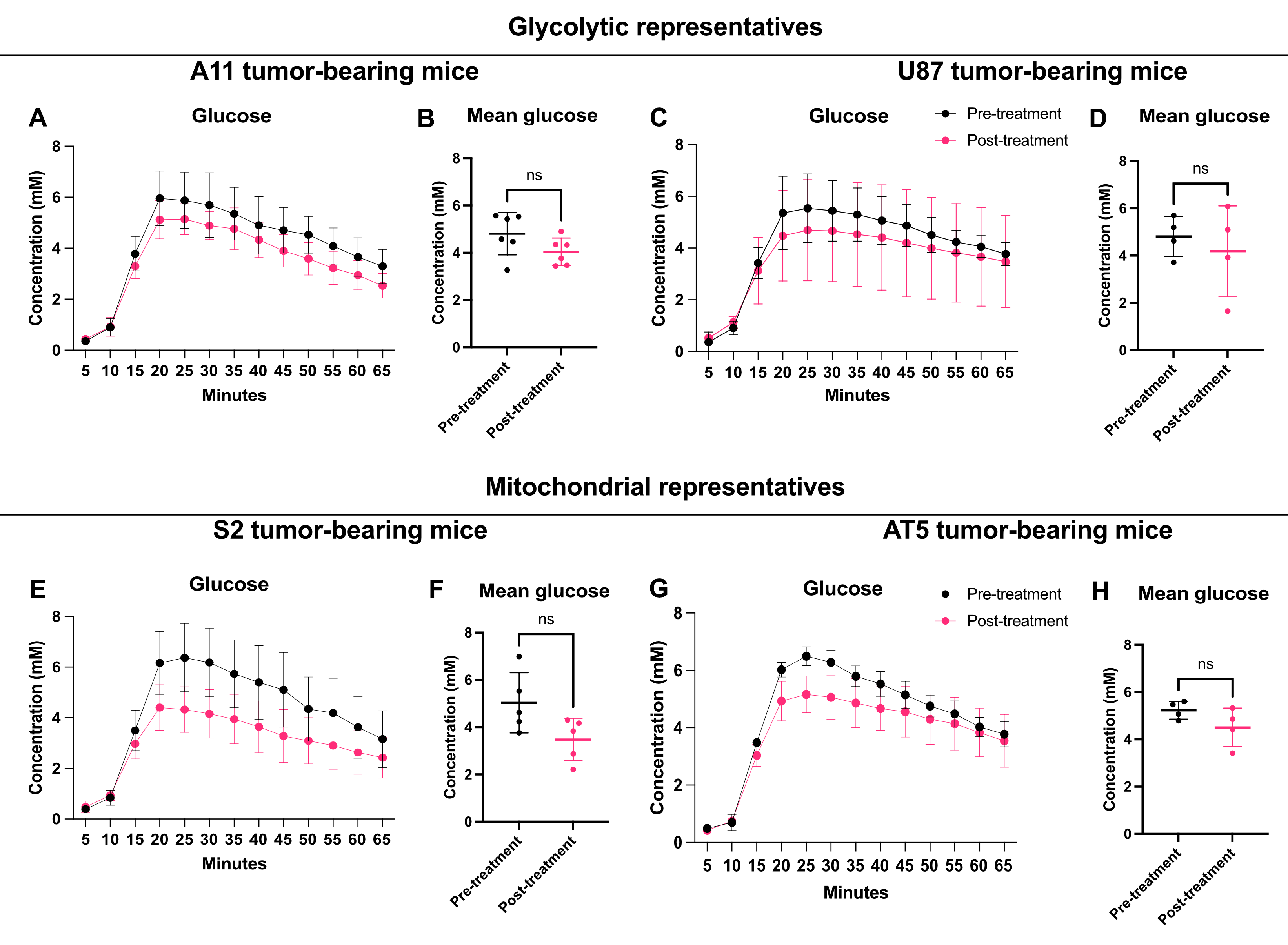


**Supplementary Figure 8.** **Spectroscopic measurements of the effects of chemoradiation on glucose uptake in tumor models representative of the glycolytic and mitochondrial subtypes.**

Serial 5-minute coil-localized ^2^H spectra were acquired from tumors representative of the glycolytic (A11 and U87) (A-D) and mitochondrial subtypes (S2 and AT5) (E-H) before and 24 h after targeted chemoradiation. The concentrations of ^2^H-labeled glucose measured between 20 minutes and 65 minutes following 2g/kg [6,6’-^2^H_2_]glucose injection were compared pre- and posttreatment in A11 (B), U87 (D), S2 (F), and AT5 (H) tumor-bearing animals. ns, not significant.


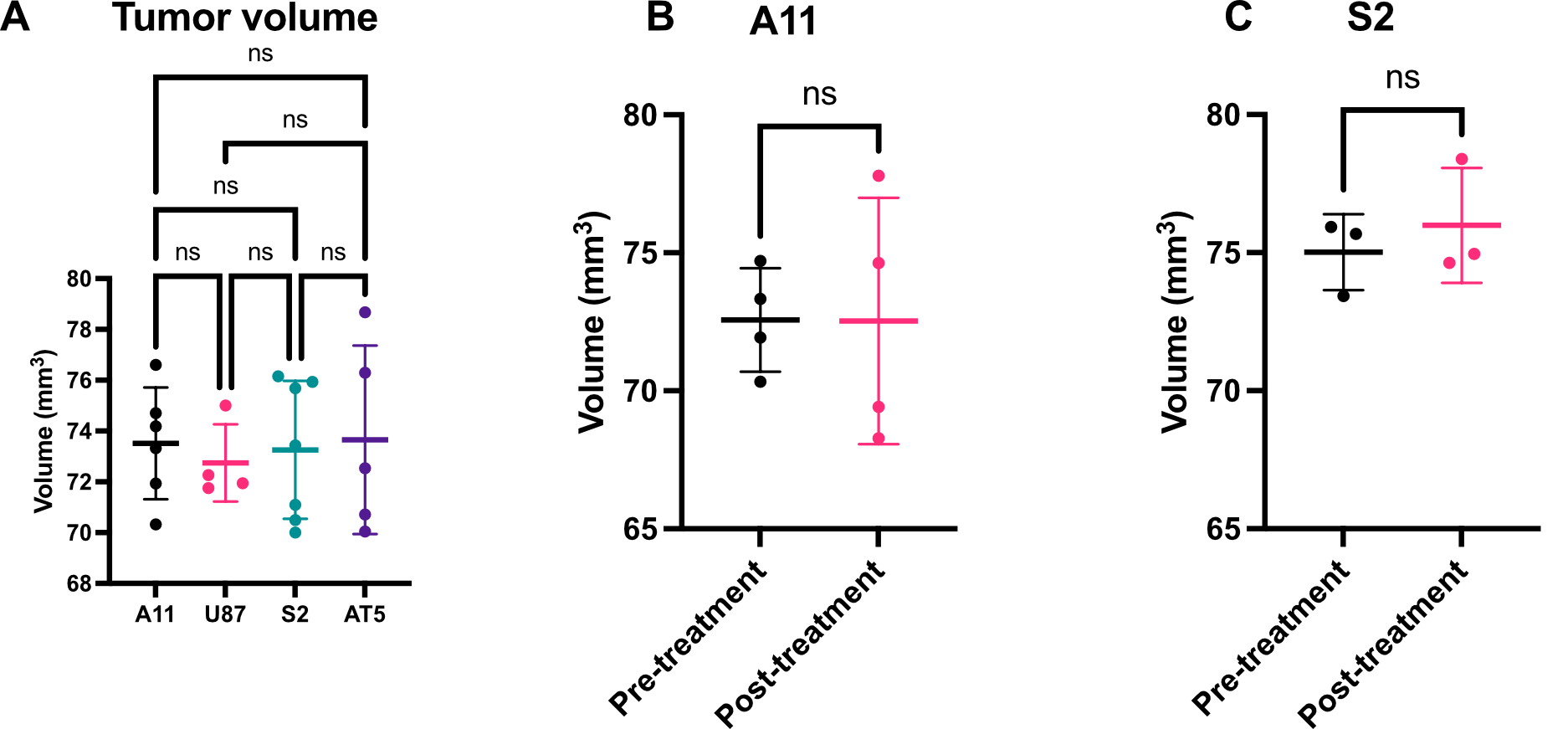


**Supplementary Figure 9. Tumor volume measurements for tumors representative of the different metabolic subtypes (A) and a comparison of A11 (B) and S2 (C) tumor volumes before and after chemoradiation.**

There was no significant differences in tumor volumes between the tumor subtypes and no change in tumor volumes 24 h after of completion of chemoradiation. ns, not significant.


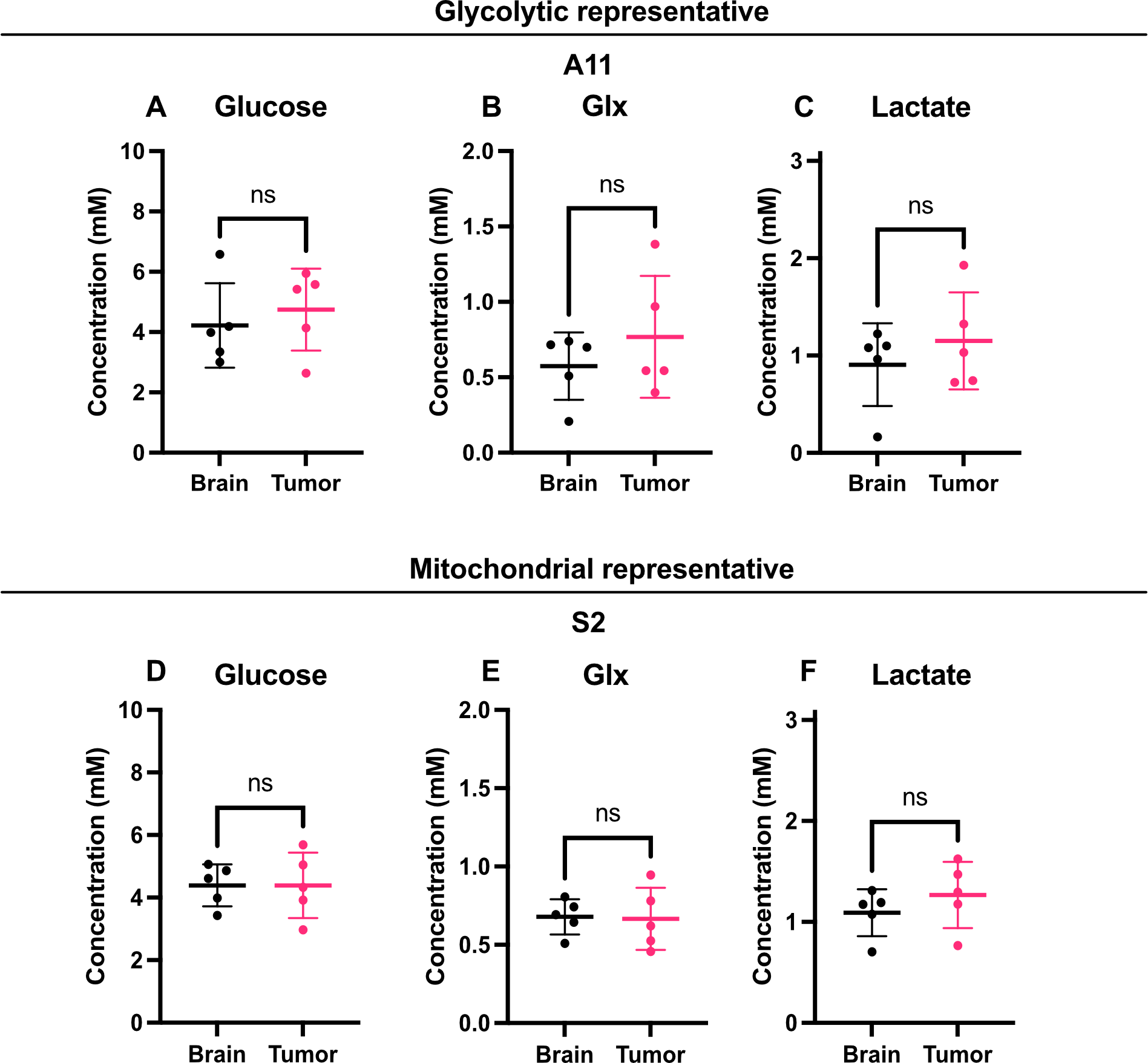


**Supplementary Figure 10. 3D ^2^H CSI of [6,6-^2^H_2_]glucose metabolism in A11 and S2 tumors and in normal appearing brain following treatment with chemoradiation.**

Comparison of metabolite concentrations in treated A11 tumors and adjacent normal appearing brain measured over a period of 60 min after injection of 2g/kg [6,6’-^2^H_2_]glucose; ^2^H-labeled glucose (A), Glx (B), and lactate (C), and in treated S2 tumors and adjacent normal appearing brain; ^2^H-labeled glucose (D), Glx (E), and lactate (F). ns, not significant.


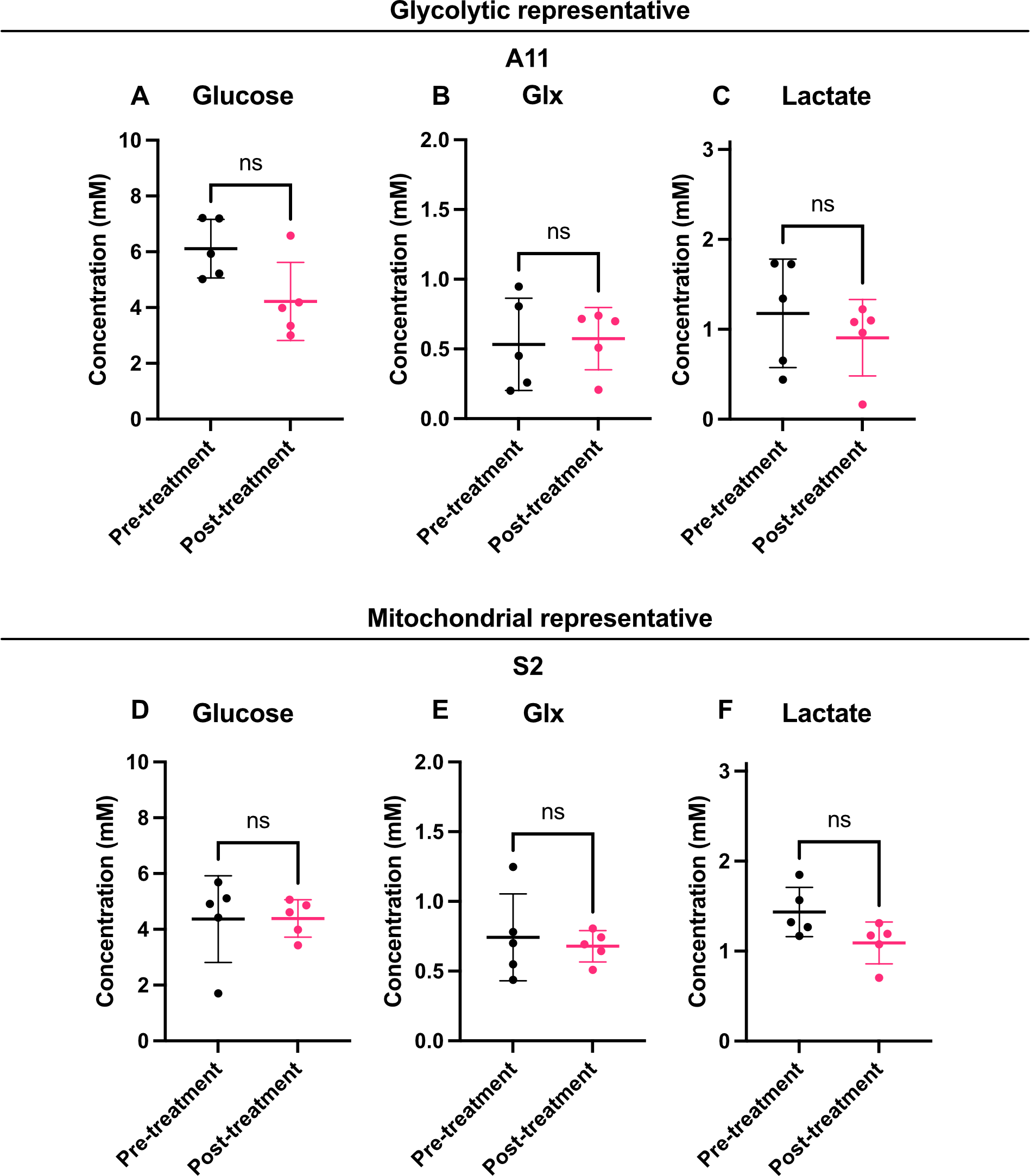


**Supplementary Figure 11. 3D ^2^H CSI of [6,6-^2^H_2_]glucose metabolism in the normal appearing brain of A11 and S2 tumor-bearing mice before and after chemoradiation.**

Comparison of metabolite concentrations in normal appearing brain in animals implanted with A11 tumors measured over a period of 60 min after injection of 2g/kg [6,6’-^2^H_2_]glucose; ^2^H-labeled glucose (C), Glx (D), and lactate (E), and in normal appearing brain in animals implanted with S2 tumors; ^2^H-labeled glucose (F), Glx (G), and lactate (H) before and after chemoradiation. ns, not significant; *p <0.05.

References

1. Garofano L, Migliozzi S, Oh YT, D'Angelo F, Najac RD, Ko A*, et al.* Pathway-based classification of glioblastoma uncovers a mitochondrial subtype with therapeutic vulnerabilities. Nat Cancer **2021**;2:141-56

2. Fala M, Ros S, Sawle A, Rao JU, Tsyben A, Tronci L*, et al.* The role of branched-chain aminotransferase 1 in driving glioblastoma cell proliferation and invasion varies with tumor subtype. Neurooncol Adv **2023**;5:vdad120
